# A Robust Method for Assaying the Immunoreactive Fraction in Nonequilibrium Systems

**DOI:** 10.3390/ph12040177

**Published:** 2019-12-03

**Authors:** Thibaut Denoël, Luca Pedrelli, Giuseppe Pantaleo, John O. Prior

**Affiliations:** 1 Department of Nuclear Medicine and Molecular Imaging, Lausanne University Hospital and University of Lausanne, Rue du Bugnon 46, 1011 Lausanne, Switzerland; luca.pedrelli@alumni.epfl.ch (L.P.); john.prior@chuv.ch (J.O.P.); 2 Division of Immunology and Allergy, Department of Medicine, Lausanne University Hospital and University of Lausanne, Rue du Bugnon 46, 1011 Lausanne, Switzerland; giuseppe.pantaleo@chuv.ch

**Keywords:** translational pharmacology, immunoreactivity, radiopharmaceuticals, radiolabeled antibodies, binding sites, antibody, immunoconjugates, Lindmo plot

## Abstract

The immunoreactive fraction *r* provides important information on the functional purity of radiolabeled proteins. It is traditionally determined by saturating the radioimmunoconjugate with an increasing excess of antigen, followed by linear extrapolation to infinite antigen excess in a double inverse “Lindmo plot”. Although several reports have described shortcomings in the Lindmo plot, a systematic examination is lacking. Using an experimental and simulation-based approach, we compared—for accuracy, precision and robustness—the Lindmo plot with the “rectangular hyperbola” extrapolation method based on the Langmuir model. The differences between the theoretical and extrapolated *r* values demonstrate that nonequilibrium and antigen depletion are important sources of error. The mathematical distortions resulting from the linearization of the data in the Lindmo plot induce fragility towards stochastic errors and make it necessary to exclude low bound fractions. The rectangular hyperbola provides robust and precise *r* estimates from raw binding data, even for slow kinetics.

## 1. Introduction

Three common methods for radiolabeling monoclonal antibodies or proteins involve the direct radioiodination of tyrosine residues, the reaction of maleimide with cysteine and the conjugation of activated ester-or isothiocyanate-bearing chelators with lysine. In these cases, it is typically not possible to achieve selective labeling of a specific reaction site (e.g., tyrosine, cysteine or lysine) in the primary structure. This lack of selectivity results in complex heterogeneous mixtures of radiolabeled macromolecules. Often, a site in the complementarity-determining regions (CDRs) of antibodies is radiolabeled, leading to the disruption of the binding affinity. In addition, oxidative damage may occur in the primary structure owing to the reaction conditions or to radiolysis. Since structural similarities make separation impossible by chromatography, the resulting non-immunoreactive impurities are present in the final product. Therefore, quantification of the percentage of immunoreactive radioactive compound relative to the total radioactive mixture is required. This test is an immunoreactivity assay, which is currently a mandatory step in the development of new radiolabeled monoclonal antibodies [1,2,3,4].

There is no established method for determining the immunoreactive fraction (*r*). Ideally, *r* is equal to the maximum value of the specifically bindable radioactive antibody, divided by the total radioactivity. Over the years, several methods have been proposed to give an estimate of *r*: one-point determination [5,6,7], multi-points assays with concentration variation in antibody [8], antigen [9], or both antibody and antigen [10], saturation assays with excess antigen [11], or kinetic assays [12]. Several physical supports for antigens have been used: live or fixed cells in tubes [6,11], coated microplates [13], coated beads [7,14], cell membranes on filter plates [10], and immunoaffinity columns [15]. Experimentally, *r* can be defined as the maximal bound fraction attained [5,6,15]; alternatively, the data points are mathematically processed by extrapolation of the binding isotherm: (i) as a function of antigen [8,16], (ii) as a function of radiolabeled antibody [8,16], or (iii) by multi-variables iterative computation [10,17]. By far, the most commonly used method for the determination of *r* is that proposed by Lindmo et al. in 1984, which involves a saturation assay with an excess of antigen followed by data linearization and extrapolation of *r* to infinite antigen excess [11].

To our knowledge, whilst the intricacies of radioimmunoassays describing the pharmacological properties of drugs (i.e., *K_D_*, IC_50_, *k_off_*, etc.) have been extensively discussed [10,18,19,20,21], the critical aspects specifically concerning the immunoreactive fraction assay (IRFA) have not been systematically analysed. For example, achieving the necessary dynamic equilibrium is generally taken for granted in IRFA and their low reproducibility is attributed to a lack of sufficient antigen excess [8,9,11,22]. Furthermore, the poor error management—especially at low bound fractions—resulting from the linear transformation used by Lindmo et al., has not been discussed [11].

In this paper, we systematically analysed the theoretical basis of an IRFA. After examining the binding curves, the main assumptions, the experimental context, the error propagation, and the kinetics, randomness was introduced to simulate an experimental setting. Our simulation compares two approximate equations for the extrapolation of *r* and *K_D_*, namely the conventional saturation binding curve (rectangular hyperbola) and the Lindmo plot (linear transformation). Examples are given with radiolabeled antibodies displaying slow kinetics. It will become evident that the method of Lindmo et al. leads to chaotic behaviour because of errors amplification at low binding. Instead, we propose a more precise and robust method based on the Langmuir isotherm to obtain the immunoreactive fraction of radioimmunoconjugates from complete binding data. This method will prove more useful for future evaluation of new imaging and theranostic radiopharmaceuticals.

## 2. Methods

### 2.1. General Equation from the Law of Mass Action

The simplest model for the binding of an antibody (*Ab*) to an antigen (*Ag*) postulates monovalent binding [19]. Then, the antigen-antibody complex 1:1 (*Ag•Ab*, also called *B*) is the product of the association reaction:

(1) $$Ag+Ab\overset{k_{on}}{\to }B$$

The association reaction follows a rate of second order proportional to the free concentration of antigen and antibody—[*Ag*] and [*Ab*]—with the association rate constant *k_on_*:

(2) $$v_{on}=k_{on}\cdot \left[Ag\right]\cdot \left[Ab\right]$$

The dissociation of the antigen-antibody complex into starting components is the reverse reaction:

(3) $$B\overset{k_{off}}{\to }Ag+Ab$$

The first order rate for the dissociation of the *B* complex depends on the concentration of *B* by the dissociation rate constant *k_off_*:

(4) $$v_{off}=k_{off}\cdot \left[B\right]$$

When the dissociation rate of the complex is equal to its association rate, the system is in dynamic equilibrium:

(5) $$k_{on}\cdot \left[Ag\right]\cdot \left[Ab\right]=k_{off}\cdot \left[B\right]$$

The law of mass action defines the equilibrium dissociation constant (*K_D_*) of the system as follows:

(6) $$K_{D}=\frac{k_{off}}{k_{on}}=\frac{\left[Ag\right]\cdot \left[Ab\right]}{\left[B\right]}$$

The concentration of the antigen-antibody complex at equilibrium is

(7) $$\left[B\right]=\frac{\left[Ag\right]\cdot \left[Ab\right]}{K_{D}}$$

If the total applied antibody (*T*) contains a non-reactive damaged antibody, only a fraction of *T* is immunoreactive. Let *r* be the immunoreactive fraction. The concentration of free antibody [*Ab*] is the concentration of the total reactive antibody [r·T] minus the bound antibody [*B*], and

(8) $$\left[B\right]=\frac{\left[Ag\right]\cdot \left[r\cdot T-B\right]}{K_{D}}$$

The equation, which is a modification of the Langmuir isotherm, is reorganized as follows:

(9) $$\frac{\left[B\right]}{\left[T\right]}=r\cdot \frac{\left[Ag\right]}{\left[Ag\right]+K_{D}}$$

### 2.2. Approximate Equations for the Immunoreactive Fraction Assay

In this assay, the bound fraction $\frac{\left[B\right]}{\left[T\right]}$ of the labeled antibody is a function of the concentration of free antigen [*Ag*], the immunoreactive fraction *r* and the dissociation constant *K_D_*. For [*Ag*] ≫ *K_D_*, the bound fraction $\frac{\left[B\right]}{\left[T\right]}$ at equilibrium approaches the immunoreactive fraction *r*.

During the assay, [*Ag*] is unknown, however, in the presence of a large excess of antigen, the [*Ag*] concentration can be approximated with the total concentration of antigen [*Ag*]*_Total_*. At the beginning of the experiment (*t*_0_), [*Ag*]_0_ = [*Ag*]*_Total_* and at equilibrium:

(10) $$\frac{\left[B\right]}{\left[T\right]}≅r\cdot \frac{{\left[Ag\right]}_{Total}}{{\left[Ag\right]}_{Total}+K_{D}}=r\cdot \frac{{\left[Ag\right]}_{0}}{{\left[Ag\right]}_{0}+K_{D}}$$

A single-point experiment with [*Ag*]_0_ ≫ [*T*] and [*Ag*]_0_ ≫ *K_D_* will give a minimal value of *r* from the bound fraction $\frac{\left[B\right]}{\left[T\right]}$. For greater accuracy, a conventional saturation assay may be performed with several increasing concentrations of antigen until $\frac{\left[B\right]}{\left[T\right]}$ reaches a maximum. The series of data points can be fitted with *K_D_* and *r* as parameters in a rectangular hyperbolic binding curve [23]. This isotherm of the bound fraction $\frac{\left[B\right]}{\left[T\right]}$ as a function of [*Ag*]_0_, extrapolated to an infinite concentration of antigen, provides the most accurate value for the immunoreactive fraction *r*. In addition, the dissociation constant *K_D_* can be extracted from the fit using any modern software.

Often, a transformation of the data points is done by a double inversion (Equation (11)). The data thus analysed is known as the Lindmo plot [11].

(11) $$\frac{\left[T\right]}{\left[B\right]}≅\frac{1}{r}+\frac{1}{{\left[Ag\right]}_{0}}\cdot \frac{K_{D}}{r}$$

A linear extrapolation of the double inverse plot of $\frac{\left[T\right]}{\left[B\right]}$ as a function of $\frac{1}{{\left[Ag\right]}_{0}}$ gives the reciprocal of the immunoreactive fraction at *y*-intercept and the slope is equal to $\frac{K_{D}}{r}$.

### 2.3. Exact Expression of the Bound Fraction as a Second-Order Equation

In the previous section, Equation (9) was modified to use [*Ag*]_0_ instead of [*Ag*] because, by reason of antigen depletion, the concentration of free antigen is unknown except at *t_0_*. Equations (10) and (11) thus obtained are useful approximations for the assay. The correct equation linking [*Ag*]_0_, *B*, *T*, *r* and *K_D_* is developed below.

From the equalities $\left[Ag\right]={\left[Ag\right]}_{0}-{\left[Ag\right]}_{Bound}$ and ${\left[Ag\right]}_{Bound}={\left[Ab\right]}_{Bound}=\left[B\right]$, Equation (9) is expressed as follows:

(12) $$\frac{\left[B\right]}{\left[T\right]}=r\cdot \frac{{\left[Ag\right]}_{0}-\left[B\right]}{{\left[Ag\right]}_{0}-\left[B\right]+K_{D}}$$

By artificially multiplying the numerator and the denominator on the right-hand side by $\frac{1}{\left[T\right]}$:

(13) $$\frac{\left[B\right]}{\left[T\right]}=r\cdot \frac{\frac{{\left[Ag\right]}_{0}}{\left[T\right]}-\frac{\left[B\right]}{\left[T\right]}}{\frac{{\left[Ag\right]}_{0}}{\left[T\right]}-\frac{\left[B\right]}{\left[T\right]}+\frac{K_{D}}{\left[T\right]}}$$

By rearranging the terms,

(14) $$\frac{\left[B\right]}{\left[T\right]}\cdot \left(\frac{{\left[Ag\right]}_{0}}{\left[T\right]}-\frac{\left[B\right]}{\left[T\right]}+\frac{K_{D}}{\left[T\right]}\right)=r\cdot \frac{{\left[Ag\right]}_{0}}{\left[T\right]}-r\cdot \frac{\left[B\right]}{\left[T\right]}$$

Finally,

(15) $$\frac{{\left[B\right]}^{2}}{{\left[T\right]}^{2}}+\frac{\left[B\right]}{\left[T\right]}\cdot \left(-\frac{K_{D}}{\left[T\right]}-\frac{{\left[Ag\right]}_{0}}{\left[T\right]}-r\right)+r\cdot \frac{{\left[Ag\right]}_{0}}{\left[T\right]}=0$$

The quadratic equation is solved with respect to $\frac{\left[B\right]}{\left[T\right]}$ and two solutions are found:

(16) $$\frac{\left[B\right]}{\left[T\right]}=\frac{\frac{K_{D}}{\left[T\right]}+\frac{{\left[Ag\right]}_{0}}{\left[T\right]}+r\pm \sqrt{{\left(\frac{K_{D}}{\left[T\right]}+\frac{{\left[Ag\right]}_{0}}{\left[T\right]}+r\right)}^{2}-4r\cdot \frac{{\left[Ag\right]}_{0}}{\left[T\right]}}}{2}$$

One solution (+) is discarded because it is not equal to the origin when ${\left[Ag\right]}_{0}$ is equal to zero and does not converge to *r* at infinite ${\left[Ag\right]}_{0}$. The second solution (−) is kept in order to generate datasets with the theoretical binding curve. Similar equations have been described in the literature [8,10,20].

### 2.4. Goodness of Fit

A dataset of the theoretical bound fractions $\frac{\left[B\right]}{\left[T\right]}$ as a function of [*Ag*]_0_ is generated with Equation (16) (Matlab R2017a, RRID:SCR_001622). The two approximate models are fitted (GraphPad Prism 8, RRID:SCR_002798) to the obtained values. The rectangular hyperbola (Equation (10)) provides the immunoreactive fraction *r* at infinite antigen excess and *K_D_*. The Lindmo plot (Equation (11)) by least squares linear regression gives $\frac{1}{r}$ at the *y*-intercept and the slope multiplied by *r* gives *K_D_*. The extrapolated results are compared to the original values. The goodness of fit for the immunoreactive fraction *r* and the dissociation constant *K_D_* is reflected in the lack of discrepancy for a wide range of *K_D_* and [*T*] parameters. The [*Ag*]_0_ used are ${\left[Ag\right]}_{0}=\left\{1.25;2.5;5;10;20;40;80\right\}\;\mathrm{nM}$ and *r* = 70% in all cases. In doing so, the monovalent binding, the total adsorption of applied antigen on the surface of the plate, the total loss of affinity for a damaged antibody, the reversibility of complex formation and the attainment of equilibrium are assumed to be true. The tested assumptions are (*i*) [*Ag*]_0_ ≫ *K_D_*, (*ii*) [*Ag*]_0_ ≫ [*T*], (*iii*) [*T*] ≪ *K_D_*.

### 2.5. Study of the Assumption [Ag]_0_ ≫ K_D_

A common statement postulates that in IRFA, a maximum plateau of the bound fraction must be reached [22]. Let [*Ag*]_0_ ≫ [*T*]. Consider the following equation, obtained from the division of the numerator and the denominator of Equation (10) by [*Ag*]_0_:

(17) $$\frac{\left[B\right]}{\left[T\right]}=r\cdot \frac{1}{1+\frac{K_{D}}{{\left[Ag\right]}_{0}}}$$

In a single point experiment, a threshold of 91% of the value of *r* is obtained from the bound fraction $\frac{\left[B\right]}{\left[T\right]}$ if [*Ag*]_0_ ≥ 10 · *K_D_*. However, in the case of an experiment using several data points, such an excess of [*Ag*]_0_ over *K_D_* is not required for a good estimate of the value of *r*. By definition, the equation of a rectangular hyperbola is completely determined by two points and the origin. Therefore, any two $\frac{\left[B\right]}{\left[T\right]}$ values as a function of [*Ag*]_0_ (even those of the steep part of the curve if the *K_D_* is high) will provide a correct estimate of *r* at infinite [*Ag*]_0_. In the same way, using the Lindmo plot, any two $\frac{\left[T\right]}{\left[B\right]}$ values as a function of $\frac{1}{{\left[Ag\right]}_{0}}$ will give $\frac{1}{r}$ at the *y*-intercept and $\frac{K_{D}}{r}$ from the slope. Consequently, in theory, it is not necessary to have *K_D_* ≪ [*Ag*]_0_, nor to reach a plateau of the bound fraction. Computational verification involves a dataset of original $\frac{\left[B\right]}{\left[T\right]}$ values as a function of [*Ag*]_0_, for a set of *K_D_*, at a fixed [*T*] = 0.1 nM. Values for *K_D_* cover four orders and are $K_{D}=\left\{0.01;0.0316;0.1;0.316;1;3.16;10;31.62;100\right\}\;\mathrm{nM}$.

### 2.6. Study of the Assumptions [Ag]_0_ ≫ [T] and [T] ≪ K_D_

The assumption of [*T*] ≪ *K_D_* is described as important in the literature [16]. The assumption [*Ag*]_0_ ≫ [*T*] is required to approximate [*Ag*] with [*Ag*]_0_. Mathematical verification is performed from a set of original $\frac{\left[B\right]}{\left[T\right]}$ values as a function of [*Ag*]_0_, for several [*T*], at a fixed *K_D_* (1 nM). Ten total antibody concentrations [T]={0.0061;0.0183;0.0549;0.165;0.494;1.48;4.44;13.3;40;120} nM are tested. Graphs are provided to visually assess goodness of fit for the individual data points.

Graphs analysing in parallel the assumptions [*T*] ≪ *K_D_* and [*Ag*]_0_ ≫ [*T*] on the extrapolation of *r* and *K_D_* are useful for studying the effect of antigen depletion with various *K_D_* values. In this way, the estimated *r* and *K_D_* are shown for the four $K_{D}=\left\{0.01;0.1;1;10\right\}\;\mathrm{nM}$ as a function of $\frac{\left[T\right]}{{\left[Ag\right]}_{0,min}}$. The concentration [*Ag*]_0,*min*_, is the minimum concentration of total antigen from the vector of [*Ag*]_0_ (i.e., 1.25 nM).

A laboratory experiment using the CD4bs anti-gp120 ^177^Lu-PCTA-VRC07 antibody bound to the 426c core gp120 heptamer provides a comparison for the extrapolation of *r* and *K_D_* at increasing [*T*].

### 2.7. Antigen, Antibodies and Radiolabeling

The recombinant protein 426c core gp120 heptamer is a modified envelope (Env) glycoprotein derived from a primary clade C virus (426c) isolated from an HIV-1 infected subject. The variable regions 1, 2 and 3 were deleted and three *N*-linked glycosylation sites in loop D and in variable region 5 of 426c gp120 were removed to allow binding to germline B cell receptors [24]. Heptamerisation was achieved by adding a 55 amino acids peptide from the multimerisation domain of the human C4b-binding protein to the carboxy terminus of 426c Core. This antigen of molecular weight 315 kDa binds to the VRC01-class antibodies directed against the CD4-binding site (CD4bs) of the HIV-1 Env gp120 including VRC01, VRC07, 3BNC117.

The human (IgG1) monoclonal antibody 3BNC117 belongs to a recently discovered family of broadly neutralising monoclonal antibodies (bnMAbs) directed to the CD4bs of the HIV-1 Env protein gp120. The 3BNC117 antibody was isolated from a HIV-1 positive donor and has been produced in a Chinese Hamster Ovary (CHO) cell line CHOK1.SV. The 3BNC117 antibody has been shown to block about 90% of HIV isolates [25]. The human (IgG1) monoclonal antibody VRC07 is a variant of the bnMAb VRC01. VRC07 is also directed to the CD4bs of gp120 and neutralizes 96% of HIV isolates [26].

These two antibodies binding to gp120 were used to provide illustrative examples of two different radiolabeling methods, namely a direct radioiodination and a conjugation-chelation process. Radioiodination involves short oxidative stress due to the presence of the iodogen chlorinating agent and target tyrosine. Conjugation involves a long basic stress (pH 9.5) and target lysine. The conjugated antibody can be chelated later with different metal radioisotopes.

Radioiodination of 3BNC117 was done in a glass vial coated with 50 μg iodogen by the addition of a phosphate buffer solution (PO_4_^3−^, 0.2 M, pH 8.0, 125 μL), 3BNC117 (100 μg, 100 μL) and 5 MBq of sodium ^125^I-iodide (Perkin Elmer, specific activity of 629 GBq/mg, 30 μL). After 5 min, the reaction was recovered by aspiration and quenched with 300 μL of a 2% ascorbate in PO_4_^3−^ buffer. The unreacted ^125^I-iodide was removed by ultrafiltration with PO_4_^3−^ buffer. ^125^I-3BNC117 was obtained with a purity of >95% (iTLC, citrate buffer pH 5; HPLC, XBridge Protein BEH SEC 200Å, PO_4_^3−^ buffer 0.1 M with 2% NaCl, pH 6.5).

The conjugation of VRC07 (334 µg) was realized after buffer exchange using ultrafiltration (Amicon Ultra, 0.5 mL, 50 kD) with four successive additions of carbonate/bicarbonate buffer (CBC, 0.4 M, pH 9.5). A 14.2 µL volume (20 eq.) of a freshly made solution of *p*-SCN-Bn-PCTA (Macrocyclics, 1.0 mg) in DMSO (50 µL) and CBC (0.4 M, pH 9.5, 0.450 mL) was added to the concentrated VRC07 solution and stirred at RT for 16 h. The excess chelator was removed by ultrafiltration with four NaCl 0.9% additions. The 40 µL solution was stored at the approximate concentration of 8 mg/mL. PCTA-VRC07 was homogeneous in HPLC (XBridge Protein BEH SEC 200Å, 3.5 μm, 7.8 × 300 mm Waters, PO_4_^3−^ buffer 0.1 M with 2% NaCl, pH 6.5) and had preserved binding to gp120-expressing tumour cell line HeLa 243 (flow cytometry). Radiolabeling was realized with ^177^LuCl_3_ (EndolucinBeta, specific activity of 3500 GBq/mg). To 150 µL of NH_4_OAc/AcOH buffer (pH 5.4, 0.4 M), 12.5 µL of PCTA-VRC07 (8 mg/mL, 100 µg) and 20 MBq of diluted ^177^Lu (10 µL) were added. After 1 h of incubation at 37 °C, ^177^Lu-PCTA-VRC07 was obtained without further purification with a purity of 98% (iTLC, citrate buffer pH 5).

### 2.8. Immunoreactive Fraction Assay on Microplate

The assay uses an antigen coated on a high-binding microplate in polystyrene (PS) (Corning 96 Well EIA/RIA Clear Flat Bottom Polystyrene High Bind Microplate, 360 µL). In this mathematical model, we assume a 100 kDa univalent antigen that is completely adsorbed on the plate and has fully accessible binding sites. A triplicate of 100 µL of antigen in PBS at concentrations ${\left[Ag\right]}_{0}=\left\{1.25;2.5;5;10;20;40;80\right\}\;\mathrm{nM}$ (antigen molecular weight e.g., 100 kDa) was coated for 16 h at 5 °C. The uncoated antigen was decanted by flicking the plate; the wells were rinsed and blocked with 2% BSA for 2 h. Three empty wells were also blocked for nonspecific binding determination. The wells were rinsed with 2% BSA and 100 µL of a 0.1 nM solution (0.015 µg/mL for an antibody molecular weight of e.g., 150 kDa) of freshly labeled antibody in 2% BSA were added. The concentration of this solution is known from HPLC measurement post-labeling and standard dilution techniques. The same quantity was also placed in three γ-counter tubes for the measurement of the total activity *T* (internal control). The incubation time and temperature depend on the previous kinetic experiments, the stability of the radioimmunoconjugate and the half-life of the radioisotope, for example, 20 h of incubation at 5 °C. The unbound antibody (*U*) present in the supernatants of each well was pipetted into γ-counter tubes, the wells were rinsed once with cold PBS and the pooled fractions were counted (in case of high *k_off_*, this rinse is cancelled). The specifically bound antibody (*B_Spec_*) was calculated by the following equation, which takes into account nonspecific binding (*B_NS_*):

(18) $$B_{Spec}=T-U-B_{NS}$$

The bound fraction in the blank wells (wells blocked but not coated with antigen) is nonspecific and corresponds to

(19) $$0=T-U_{Blank}-B_{NS}$$

Nonspecific binding is invariant in this assay because the coated PS surface area is invariant, its value is

(20) $$B_{NS}=T-U_{Blank}$$

It follows logically that

(21) $$B_{Spec}=U_{Blank}-U$$

In the Supplementary Information, a step by step protocol for the immunoreactive fraction assay of radiolabeled antibodies using coated antigens on microplate is presented.

### 2.9. Kinetic Assay on Microplate

For kinetic experiments, two plates were run in parallel as above but with six rows each and each row contains three copies of blank wells. All manipulations were performed on ice and incubation at 5 °C. After the application of the labeled antibody, a counter was started. A row (without replicates) with the corresponding triplicate of blank wells were pipetted into γ-counter tubes exactly at times t={15;30;60;120;240;960} min. From the second plate, duplicate wells were pipetted into tubes at times t={1440;2400;2880} min with the respective blank triplicate. Wells were not rinsed.

### 2.10. Error Propagation for Experimental Variations

To test the influence of stochastic errors on the goodness of fit, the original $\frac{\left[B\right]}{\left[T\right]}$ dataset for several *K_D_*, with [*T*] = 0.1 nM, *r* = 70% and ${\left[Ag\right]}_{0}=\left\{1.25;2.5;5;10;20;40;80\right\}\;\mathrm{nM}$ obtained with the theoretical binding curve (Equation (16)) was modified by simulated variations as would be obtained in an experimental setting. Stochastic variations were modelled as a quadratic sum of four errors (on ${\left[Ag\right]}_{0}$, on *T*, on $B_{NS}$ and on γ-count). For each error, a standard deviation (SD) matrix was calculated. To calculate each SD, all other errors were assumed to be zero. Notwithstanding variations, it is always assumed for error propagation that all unbound antibodies are correctly accounted for (*U* is exactly quantified). The relevant certainties for each considered error are summarized in Table 1. The systematic errors of incomplete achievement of equilibrium and saturation of the PS plate are detailed later on.

For reading purposes, in the following sections, all variables (*B*, *T*, *U*) are expressed as representative of a quantitative radioactivity value, without unit.

### 2.11. Stochastic Variations in the Amount of Antigen

The standard deviation matrix for variations on coated ${\left[Ag\right]}_{0}$ was computed by first generating three matrices of theoretical $\frac{B}{T}$ data at different concentrations of total antigen. It was assumed that the whole antigen present in the applied solution was coated on the plate, within the limits of the stochastic variations. The original dataset, a set with a 10% negative difference on the antigen (${\left[Ag\right]}_{0}=\left\{1.125;2.25;4.5;9;18;36;72\right\}\;\mathrm{nM}$), and another with a 10% positive difference on the antigen (${\left[Ag\right]}_{0}=\left\{1.375;2.75;5.5;11;22;44;88\right\}\;\mathrm{nM}$) were constructed. This percentage was chosen to approximate serial dilution errors and variations in binding uniformity for a microplate [27]. Assuming a normal distribution, the three matrices were used to calculate the SD matrix from triplets of $\frac{B}{T}$ values.

### 2.12. Stochastic Variations in the Amount of Antibody

The SD matrix for *T* variations represents uncertainties in the volume of labeled antibody pipetted into the blocked wells. A systematic error in the concentration of radiolabeled antibody solution is possible but irrelevant. The difference in volume of *T* will be reflected in the radioactivity measured in the supernatant (*U*).

In the absence of nonspecific binding, Equation (19) gives:

(22) $$T=U_{Blank}$$

Within the assay, the ${\bar{U}}_{Blank}$ value will be equated with *T* and used for all well computations. The value of *T* is assumed to be constant; however, this is not the case. Consider that for a certain well, there is a difference between the *T′* actually added and *T* equal to

(23) $$\Delta T=T^{\prime }-T$$

The bound fraction $\frac{B}{T}$ is invariant to the change in *T*, as long as [*Ag*]_0_ is in large excess. Hence,

(24) $$\frac{B}{T}=\frac{B^{\prime }}{T^{\prime }}$$

Moreover, in the absence of nonspecific binding T=B+U and $T^{\prime }=B^{\prime }+U^{\prime }$, so

(25) $$\frac{U}{T}=1-\frac{B}{T}=1-\frac{B^{\prime }}{T^{\prime }}=\frac{U^{\prime }}{T^{\prime }}$$

For that well, there is a difference $\Delta U=U^{\prime }-U$ between the measured supernatant *U′* and the supernatant *U* obtained if the constant *T* had been applied. From Equation (25),

(26) $$\Delta U=\frac{U\cdot T^{\prime }}{T}-U=\frac{U\cdot T^{\prime }-U\cdot T}{T}$$

Therefore,

(27) $$\Delta U=\frac{\Delta T}{T}\cdot U$$

In the assay, *T′* is not measured; instead, $T={\bar{U}}_{blank}$ is substituted for *T′*. The resulting apparent unbound fraction $\frac{U^{\prime }}{T}$ is equal to

(28) $$\frac{U^{\prime }}{T}=\frac{U+\Delta U}{T}=\frac{U+\frac{\Delta T}{T}\cdot U}{T}$$

The apparent bound fraction can be expressed as follows:

(29) $$\frac{B^{\prime }}{T}=1-\frac{U^{\prime }}{T}=1-\left(\frac{U}{T}+\frac{\Delta T}{T}\cdot \frac{U}{T}\right)$$

Finally, from Equation (25):

(30) $$\frac{B^{\prime }}{T}=\frac{B}{T}-\frac{\Delta T}{T}\cdot \left(1-\frac{B}{T}\right)$$

To evaluate the error introduced by changes in *T*, three matrices of theoretical $\frac{B}{T}$ data are constructed, one with the original $\frac{B}{T}$ values and the other two with $\frac{B^{\prime }}{T}$ transformed by the Equation (30) for an error $\frac{\Delta T}{T}$ of ± 1%. This error percentage is chosen to approximate the imprecision of a micropipette with a viscous liquid [28]. Assuming a normal distribution, the three matrices are used to calculate the SD matrix from triplets of $\frac{B}{T}$ values.

### 2.13. Stochastic Variations in the Nonspecific Binding

Variations in nonspecific binding account for the nonspecific binding difference between blanks and coated wells. In case of nonspecific affinity of the radiolabeled antibody with the PS surface of a well, this nonspecific binding must be subtracted from the bound fraction. In each well, the bound antibody is

(31) $$B=B_{Spec}+B_{NS}$$

Total antibody *T* is the sum of unbound, specific binding and nonspecific binding, so that

(32) $$T=U+B_{Spec}+B_{NS}$$

By definition, for the blanks, there is only nonspecific binding:

(33) $$T=U_{Blank}+B_{NS}$$

Since *T* is constant (Table 1) and *U* is exactly quantified, in case of uncertainty in the nonspecific binding such that $B_{NS}^{\prime }=B_{NS}\pm \Delta B_{NS}$, the difference $\Delta B_{NS}$ is reflected on $U_{Blank}$ and

(34) $$\Delta B_{NS}=\Delta U_{Blank}$$

More generally, in the absence of error on $B_{Spec}$, the difference in the nonspecific binding gives an equal difference on *U*:

(35) $$\Delta B_{NS}=\Delta U$$

Since the amount of antibody *T* is constant, Equations (32) and (33) can be equated:

(36) $$U+B_{Spec}+B_{NS}=U_{Blank}+B_{NS}$$

Because the coated PS surface area is the same for all the wells, the mean value of the nonspecific binding $\bar{B_{NS}}$ is constant and the nonspecific binding can be simplified:

(37) $$U+B_{Spec}=U_{blank}$$

Equation (21) is obtained.

However, although $\bar{B_{NS}}$ can be simplified from Equation (36), the differences $\Delta B_{NS}$ have to be accounted for. According to Equations (21), (34) and (35), the error that will be propagated to $B_{Spec}$ is equal to

(38) $$\Delta B_{Spec}=\sqrt{{\left(\Delta U_{blank}\right)}^{2}+{\left(\Delta U\right)}^{2}}=\sqrt{2}\cdot \Delta B_{NS}=\sqrt{2}\cdot \Delta U_{blank}$$

The SD of the bound antibody imputable to nonspecific binding changes is estimated at $\sqrt{2}\cdot \Delta U_{blank}$. The $\Delta U_{blank}$ value is determined as a SD value from a set of laboratory experiments.

### 2.14. Gamma Counter Errors

The measurement of the radioactivity of the wells relies on a γ-counter, a source of experimental errors. Various causes for errors in the counting step have been discussed in the literature, e.g., geometric errors and random noise [29]. Geometric errors can be minimized by using the same tubes for all samples and the same volume of liquid. The error introduced by the random noise is important for the low activities (<1000 cpm) often used in IRFA. These come from a low specific activity of radiolabeled antibodies and the need for [*T*] ≪ [*Ag*]_0_. The error can be minimized by using a higher specific activity or a higher [*T*] value. The signal-to-noise ratio (SNR) can also be improved by averaging the measurements over a longer period due to the SNR dependence on the square root of the acquisition time. In our simulation, the standard deviation of the bound antibody attributable to the error of the γ-counter is constant and estimated at $0.01\cdot U_{blank}$.

### 2.15. Overall Stochastic Errors

Assuming a normal distribution, the previously developed SD matrices for stochastic errors on antigen quantity and amount of antibody are summed with the constant SD values for nonspecific binding changes and for γ-counter error to obtain a matrix of overall SD. The quadratic sum of the four standard deviations is equal to

(39) $$\frac{\left[\Delta B\right]}{\left[T\right]}=\sqrt{{\left(\frac{{\left[\Delta B\right]}_{Antigen}}{\left[T\right]}\right)}^{2}+{\left(\frac{{\left[\Delta B\right]}_{Antibody}}{\left[T\right]}\right)}^{2}+{\left(\frac{{\left[\Delta B\right]}_{Non-Specific}}{\left[T\right]}\right)}^{2}+{\left(\frac{{\left[\Delta B\right]}_{\gamma -Counter}}{\left[T\right]}\right)}^{2}}$$

The resulting coefficients of variation (CV) are also calculated by dividing the matrix of overall standard deviations by the matrix of original $\frac{\left[B\right]}{\left[T\right]}$ data (Equation (16)). To provide more accurate interpolation of the CV, additional points were calculated at $K_{D}=\left\{20;50;65;80\right\}\;\mathrm{nM}$. Contour plots are used for visualisation based on *K_D_* and [*Ag*]_0_ (Surfer version 16, Golden Software, Golden, CO, USA).

### 2.16. Influence of Kinetics

Failure to achieve equilibrium is known to alter the results of binding assays [20]. Indeed, the equations of the binding curve (Equations (9) and (16)) and its approximations (Equations (10) and (11)) all postulate equilibrium at all data points. A thorough simulation should examine the nonattainment of equilibrium as an effect of slow binding kinetics.

For simplicity, let the complex *B* formation be irreversible in this discussion owing to negligible dissociation. Recalling the second-order association rate of the antigen-antibody complex 1:1 (Equation (2)).

Let [*Ag*]_0_ ≫ [*T*], in this case the second-order reaction $Ag+Ab\overset{k_{on}}{\to }B$ can be considered as a pseudo-first order reaction because $\left[Ag\right]≅{\left[Ag\right]}_{0}$ is invariant in the whole process. A new pseudo-first order rate constant including the starting antigen $k_{on}^{\prime }=k_{on}\cdot {\left[Ag\right]}_{0}$ can be formulated. The new rate for the association reaction is as follows:

(40) $$v_{on}≅k_{on}\cdot {\left[Ag\right]}_{0}\cdot \left[Ab\right]=k_{on}^{\prime }\cdot \left[Ab\right]$$

By integrating the differential form of the rate law $v_{on}=-\frac{\delta \left[Ab\right]}{\delta t}$, we obtain the following equation:

(41) $${\left[Ab\right]}_{t}={\left[Ab\right]}_{0}\cdot e^{-k_{on}^{\prime }\cdot t}$$

At the beginning of the reaction, ${\left[Ab\right]}_{0}$ is equal to the total concentration of immunoreactive antibody [r·T]. At any time, the free immunoreactive antibody ${\left[Ab\right]}_{t}$ is equal to the total immunoreactive antibody minus the already bound antibody in the *B* complex:

(42) $$\left[r\cdot T-B\right]=\left[r\cdot T\right]\cdot e^{-k_{on}^{\prime }\cdot t}$$

Dividing by [*T*] and after rearrangement, the bound fraction at any time is as follows:

(43) $$\frac{{\left[B\right]}_{t}}{\left[T\right]}=r\cdot \left(1-e^{-k_{on}^{\prime }\cdot t}\right)=r\cdot \left(1-e^{-k_{on}\cdot {\left[Ag\right]}_{0}\cdot t}\right)$$

Equation (43) describes the irreversible conversion of the total immunoreactive antibody with a rate constant $k_{on}^{\prime }$ dependent on ${\left[Ag\right]}_{0}$. At start time *t*_0_, $\frac{\left[B\right]}{\left[T\right]}$ is equal to zero. Then, [*B*] increases as the free immunoreactive antibody [*Ab*] decreases with a constant half-life. In an excess of antigen, the half-time $t_{\frac{1}{2}}=\frac{\ln2}{k_{on}^{\prime }}$ of complex formation does not depend on the total concentration of antibody [*T*] but only on the total concentration of antigen ${\left[Ag\right]}_{0}$ as

(44) $$t_{\frac{1}{2}}=\frac{\ln2}{k_{on}^{\prime }}=\frac{\ln2}{k_{on}\cdot {\left[Ag\right]}_{0}}$$

The above irreversible model reaching total conversion is an approximation. In a real context, complex formation is reversible, so the equilibrium value of the fraction bound to each particular ${\left[Ag\right]}_{0}$ will eventually be obtained. In this way, to estimate the kinetic error for each *K_D_*, the matrix of original $\frac{\left[B\right]}{\left[T\right]}$ values at [*T*] = 0.1 nM is multiplied by the integrated rate function ($1-e^{-k_{on}\cdot {\left[Ag\right]}_{0}\cdot t}$) at time *t* = 1200 min [20]. From this modified dataset, the *r* and *K_D_* values are estimated using hyperbola and Lindmo plots with and without exclusion of the bound fractions at [*Ag*]_0_ ≤ 5 nM.

A representative value of the *k_on_* rate constant was determined from a kinetic assay in the laboratory using the CD4bs anti-gp120 ^125^I-3BNC117 antibody bound to 426c core gp120 heptamer. For simplicity, the effective rate constant in this assay is assumed to be $k_{on}\cdot {\left[Ag\right]}_{0}$ and the *k_off_* contribution in the integrated rate constant is omitted. The dataset modified for kinetics gives qualitative results only. The interested reader will be able to find reversible models for kinetics in the literature [10,20].

### 2.17. Computation of the Maximum [Ag]_0_

A standard 96-well high-binding PS microplate binds proteins by hydrophobic interactions and π–π stacking [30]. The binding capacity of a high-binding plate is approximately 500 ng mouse IgG/cm² and the diameter of the well is 6.4 mm [27]. The geometric computation of a cylinder with a base gives 94.7 mm^2^ of PS area for a volume of 100 µL. Therefore, about 500 ng of protein can be bound using 100 µL of solution. For a representative antigen molecular weight of 100 kDa, the maximum value of [*Ag*]_0_ is 50 nM. However, in this work, a maximum level of coated [*Ag*]_0_ = 30 nM was used to account for higher molecular weight antigens or low PS binding efficiency.

### 2.18. Using Kinetics to Study PS Saturation and B Desorption

Owing to the limited amount of antigen that can be attached to the PS surface, it is necessary to recognize when the maximum amount of [*Ag*]_0_ is coated. For this, a kinetic assay is adequate. The time-course of complex formation is illustrated using the original $\frac{\left[B\right]}{\left[T\right]}$ values for a single *K_D_* = 1 nM, at [*T*] = 0.1 nM, multiplied for each [*Ag*]_0_ by the corresponding integrated rate function ($1-e^{-k_{on}\cdot {\left[Ag\right]}_{0}\cdot t}$), at different times t={15;30;60;120;240;480;960;1440;2880} min.

The cases of an unsaturated PS plate, a plate saturated at [*Ag*]_0_ = 30 nM and a saturated plate subject to desorption of the complex *B* are presented. The time-course of complex formation in the laboratory kinetic assay of the CD4bs anti-gp120 ^125^I-3BNC117 antibody bound to 426c core gp120 heptamer is presented for comparison.

### 2.19. Time-Dependence of the Estimation of r

The evolution of extrapolated *r* values at time points t={15;30;60;120;240;480;960;1440;2880} min reflects the time-dependence of the estimate of *r* in case of unreached equilibrium. To provide the dataset for different times, the theoretical fraction bound at each [*Ag*]_0_ is multiplied by the integrated rate function at that time with the appropriate $k_{on}^{\prime }$ (*k_on_* = 0.0001444 nM^−1^min^−1^). A graph of *r* as a function of time is plotted for each *K_D_* at [*T*] = 0.1 nM. To show the influence of the extrapolation method on the resulting *r* estimate, five different fits are compared simultaneously for accuracy and robustness. These are hyperbola, Lindmo plot, Lindmo plots with exclusion of [*Ag*]_0_ = 1.25 nM, [*Ag*]_0_ ≤ 2.5 nM, [*Ag*]_0_ ≤ 5 nM. The graphs are shown for an unsaturated PS plate.

In Figure S1 and S2, the graphs of *r* as a function of time for a plate saturated at [*Ag*]_0_ = 30 nM and for a saturated plate subject to desorption of 10% of the complex *B* are represented. The vector of ${\left[Ag\right]}_{0}=\left\{1.25;2.5;5;10;20;30;30\right\}\;\mathrm{nM}$ is used, [*T*] = 0.1 nM and *r* = 70%. In this desorption model, the same multiplier of 0.9 is used for $\frac{\left[B\right]}{\left[T\right]}$ values at 30 nM, regardless of the time. In Figure S3, a graph of *r* as a function of time is also presented for the kinetic assay with the anti-gp120 ^125^I-3BNC117 antibody. To illustrate the variation of the fits with time, the data at each time point are extrapolated with a Lindmo plot (excluding [*Ag*]_0_ ≤ 5 nM) and a hyperbola (Figure S4).

### 2.20. Comparing Extrapolation Methods with Simulated Data

The original $\frac{\left[B\right]}{\left[T\right]}$ dataset as a function of [*Ag*]_0_ is calculated for *r* = 70%, [*T*] = 0.1 nM and a series of nine $K_{D}=\left\{0.01;0.0316;0.1;0.316;1;3.16;10;31.62;100\right\}\;\mathrm{nM}$ (Equation (16)). The $\frac{\left[B\right]}{\left[T\right]}$ values are corrected for kinetics at each [*Ag*]_0_ by multiplication with the integrated rate function ($1-e^{-k_{on}\cdot {\left[Ag\right]}_{0}\cdot t}$) at time *t* = 1200 min with a representative rate constant *k_on_*. Then, the standard deviations $\frac{{\left[\Delta B\right]}_{Antigen}}{\left[T\right]}$ and $\frac{{\left[\Delta B\right]}_{Antibody}}{\left[T\right]}$ are adjusted to account for the systematic variations resulting of kinetics and are summed with the other two SD (Equation (39)). Finally, *n* = 25 matrices of normally distributed random $\frac{\tilde{\left[B\right]}}{\left[T\right]}$ data points are generated with a mean equal to each $\frac{\left[B\right]}{\left[T\right]}$ value and the respective standard deviation of the quadratic sum, using the Mersenne Twister pseudo-random function (Microsoft Excel 2016, RRID:SCR_016137). In this way, 95% of the simulated $\frac{[\tilde{B]}}{\left[T\right]}$ data points are within the following confidence interval for a triplicate of experiments:

(45) $$\frac{\tilde{\left[B\right]}}{\left[T\right]}=\frac{[\bar{B]}}{\left[T\right]}\pm 2\cdot \frac{1}{\sqrt{3}}\cdot \frac{\left[\Delta B\right]}{\left[T\right]}$$

Each curve of $\frac{\tilde{\left[B\right]}}{\left[T\right]}$ as a function of [*Ag*]_0_, corresponding to a single *K_D_*, is fitted with hyperbola (Equation (10)) and Lindmo plot (Equation (11)). The process is repeated *n* times for each *K_D_*. The mean and SD of the *r* and *K_D_* estimates are calculated and compared to the actual input values in the original dataset. The *r* and *K_D_* estimates after exclusion of low bound fractions are also compared: the stepwise deletion of the $\frac{\tilde{\left[B\right]}}{\left[T\right]}$ values associated with the lowest [*Ag*]_0_ levels is followed by computation of the fit with both equations.

In the Supplementary Information, two tables of estimated *r* values for *n* = 25 matrices of normally distributed random $\frac{\tilde{\left[B\right]}}{\left[T\right]}$ data points for a saturated PS plate and for a saturated plate showing desorption of *B* are shown. The influence of PS saturation on the fit results is studied by substituting $\frac{[\tilde{B]}}{\left[T\right]}$ values at [*Ag*]_0_ = 30 nM for values at [*Ag*]_0_ = 40 nM and [*Ag*]_0_ = 80 nM (Table S1). Desorption of complex *B* is studied in the same way, but in addition by multiplying the corresponding $\frac{[\tilde{B]}}{\left[T\right]}$ values (for [*Ag*]_0_ = 30 nM) by 0.9 to simulate a 10% desorption of *B* (Table S2). The influence on the estimate of *r* of the exclusion of the duplicate bound fraction at the highest, saturated [*Ag*]_0_ is quantified.

### 2.21. Graphical Analysis of Simulated Data

Graphical analyses are displayed for *n* = 10 matrices of simulated $\frac{[\tilde{B]}}{\left[T\right]}$ data points. The mean bound fractions for the ten experiments are drawn with error bars corresponding to the SD. The fits with hyperbola and Lindmo plot are compared graphically. The resulting change in goodness of fit for the Lindmo plot after exclusion of data points corresponding to [*Ag*]_0_ ≤ 5 nM is revealed.

## 3. Results

### 3.1. Study of the Assumption [Ag]_0_ ≫ K_D_

**Figure 1:**
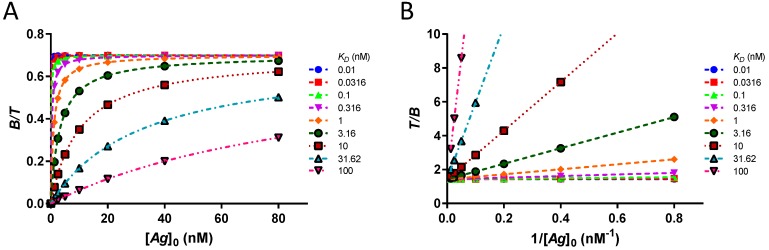
Goodness of fit for data points without error, at equilibrium and in conditions of antigen excess. (**A**) Theoretical bound fractions and approximate binding curves using the rectangular hyperbola for an antibody at [*T*] = 0.1 nM with *r* = 70%. For *K_D_* ≤ 3.16 nM, a plateau of $\frac{\left[B\right]}{\left[T\right]}$ can be qualitatively estimated when the slope tends to zero. No clear plateau is reached for *K_D_* ≥ 10 nM. Nevertheless, an excellent fit is obtained for all *K_D_* values. (**B**) The corresponding Lindmo plots use a double inversion to linearize the data. The slopes of the linear approximations are $\frac{K_{D}}{r}$. The extrapolated values of *r* from the reciprocal at *y*-intercept are accurate to 0.2%.

Using the theoretical binding curve (Equation (16)), the ideal bound fractions at equilibrium are calculated in antigen excess for an antibody with *r* = 70%. With the two approximate equations—hyperbola (Equation (10)) and Lindmo plot (Equation (11))—excellent fits are obtained (Figure 1). Estimates of *r* are accurate to 0.2% and *K_D_* to 0.1–10% with better estimates for higher *K_D_* (Table S3).

Using four orders of magnitude of *K_D_*, no clear plateau at >91% saturation is obtained for *K_D_* ≥ 10 nM (Figure 1A). The curve at *K_D_* = 100 nM does not even reach 50% saturation. Nevertheless, the extrapolated values of *r* and *K_D_* are accurate. Therefore, for ideal data points devoid of any source of error, it is not necessary to have [*Ag*]_0_ ≫ *K_D_* nor to reach a plateau. Complexes *B* with *K_D_* up to 100 nM can theoretically be used in IRFA. In the absence of antigen depletion, the hyperbola and the Lindmo plot are both excellent approximations of the theoretical binding curve at equilibrium.

### 3.2. Study of the Assumptions [Ag]_0_ ≫ [T] and [T] ≪ K_D_

**Figure 2:**
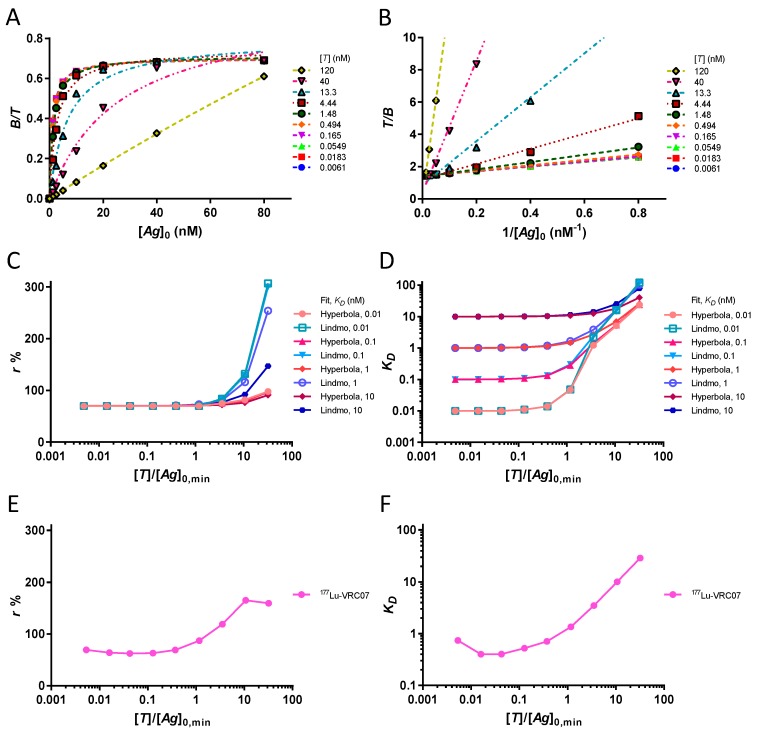
Antigen depletion adversely affects both hyperbola and Lindmo plot. (**A**) Bound fractions (points) and approximate binding curves using rectangular hyperbola at increasing [*T*] with *K_D_* = 1 nM and *r* = 70%. When [*T*] ≤ 0.494 nM, all curves overlap; [*Ag*] can be approximated to [*Ag*]_0_, and $\frac{\left[B\right]}{\left[T\right]}$ is invariant to the change in [*T*]. When [*T*] ≥ 1.48 nM, antigen depletion begins to appear as [*T*] > [*Ag*]_0,*min*_. Since [*B*] cannot be greater than [*Ag*]_0_, increasing [*T*] over [*Ag*]_0_ reduces the ratio $\frac{\left[B\right]}{\left[T\right]}$. The fitted curves extend above the plateau at $\frac{\left[B\right]}{\left[T\right]}$ = 0.7 and overestimate the value of *r*. (**B**) The corresponding Lindmo plots. The same slope equal to $\frac{K_{D}}{r}$ is observed for all [*T*] ≤ 0.494 nM and the linear fits overlap. When [*T*] ≥ 1.48 nM, antigen depletion occurs. The [*T*] exceeding [*Ag*]_0_ cannot be bound, leading to a proportional increase of the ratio $\frac{\left[T\right]}{\left[B\right]}$ at the low [*Ag*]_0_. The linear fits show increasing slopes and overestimate the value of *r*. (**C**) Variation of *r* estimates as a function of the excess of [*T*] over [*Ag*]_0,*min*_. Extrapolated *r* values are accurate as long as [*T*] < [*Ag*]_0,*min*_. When [*T*] > [*Ag*]_0,*min*_, the overestimation of *r* begins to occur and increases as [*T*] increases. The Lindmo plot provides poorer estimates of *r* under antigen depletion. (**D**) Variation of *K_D_* estimates as a function of excess of [*T*] over [*Ag*]_0,*min*_. Extrapolated *K_D_* values are accurate as long as [*T*] < [*Ag*]_0,*min*_. When [*T*] > [*Ag*]_0,*min*_, the extrapolated *K_D_* becomes proportional to [*T*]. (**C**,**D**) When [*T*] < [*Ag*]_0,*min*_, the assumption of [*T*] ≪ *K_D_* is unimportant: accurate estimates of *r* and *K_D_* are obtained for both [*T*] = 0.0061 nM with *K_D_* = 10 nM and for [*T*] = 0.494 nM with *K_D_* = 0.01 nM. (**E**) Modification of *r* values extrapolated by hyperbola of the ^177^Lu-PCTA-VRC07 antibody binding to 426c core gp120 heptamer. The experimental results show an increase in *r* estimates when [*T*] > [*Ag*]_0,*min*_. (**F**) Modification of *K_D_* values extrapolated by hyperbola of the ^177^Lu-PCTA-VRC07 complex with gp120 heptamer. When [*T*] > [*Ag*]_0,*min*_, a two orders of magnitude increase in *K_D_* values occurs. (**C**–**F**) The estimated values of *r* and *K_D_* for [*T*] = 120 nM are not given because of the disproportionate values obtained.

By varying the total antibody concentration [*T*] by several orders of magnitude, it is possible to study the conditions of depletion of the antigen. The theoretical $\frac{\left[B\right]}{\left[T\right]}$ values for increasing [*T*] are fitted with a rectangular hyperbola in Figure 2A. The corresponding Lindmo plots are drawn in Figure 2B. With both methods of approximation, the depletion of the antigen leads to an overestimation of the values of *r*. The change in *r* estimates for increasing [*T*] at four different *K_D_* values is illustrated in Figure 2C. Regardless of *K_D_*, an overestimation of *r* due to antigen depletion always appears when [*T*] > [*Ag*]_0,*min*_. A similar effect is observed for *K_D_* estimates, as shown in Figure 2D. Several orders of magnitude errors in extrapolated *K_D_* values occur when [*T*] > [*Ag*]_0,*min*_. Regarding the assumption [*T*] ≪ *K_D_*, we find that it is not relevant when [*T*] < [*Ag*]_0,*min*_ (Figure 2C,D). We confirmed, in the laboratory, the change in extrapolated *r* and *K_D_* values following antigen depletion (Figure 2E,F).

### 3.3. Stochastic Variations in the Amount of Antigen

**Figure 3:**
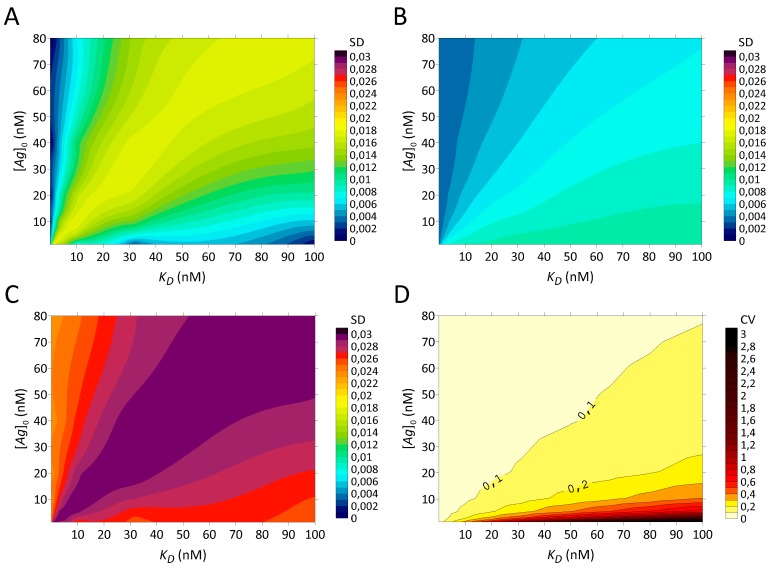
Error matrices for stochastic variations represented as contour plots of standard deviations and coefficients of variation of $\frac{\left[B\right]}{\left[T\right]}$ as a function of [*Ag*]_0_ and *K_D_*. (**A**) SD for a 10% error on [*Ag*]_0_. (**B**) SD for a 1% error on [*T*]. (**C**) Overall SD of stochastic errors. The SD of [*Ag*]_0_ and [*T*] are quadratically summed with a SD of 0.021 arising from changes in nonspecific binding and a SD of 0.01 ascribed to the count of radioactivity. The span of the overall SD is 0.023–0.030. (**D**) Overall CV of stochastic errors. The global SD are divided by their respective original bound fractions. When *K_D_* ≤ 3.16 nM or [*Ag*]_0_ > *K_D_*, the CV span 3%–10% (cream coloured area). When bound fractions decrease to 10%–20%, CV increase to 10%–30% (yellow areas). When the bound fractions become <10%, the CV increase further to overwhelm the signal with CV > 100% (red–black area at the bottom).

After examining the assumptions to obtain correct estimates of *r* and *K_D_* for theoretical data points, we can construct simulated experimental datasets. Three types of errors are considered, namely stochastic, kinetic and PS saturation. Stochastic variations are modelled as Gaussian distributions, requiring error matrices. The uncertainty on the amount of coated antigen for a 10% error on [*Ag*]_0_ lead to the standard deviation matrix represented as a contour plot in Figure 3A.

The SD range of $\frac{\left[B\right]}{\left[T\right]}$ ascribed to the error on [*Ag*]_0_ is 0.001–0.018. The SD reach their maximum when *K_D_* ≅ [*Ag*]_0_. Indeed, for [*Ag*]_0_ ≪ *K_D_*, the error on [*Ag*]_0_ leads to a relative error on a very small bound fraction, and therefore, to a smaller SD (Equation (10)). For [*Ag*]_0_ ≫ *K_D_*, because of the negligible *K_D_*, [*Ag*]_0_ vanishes, effectively removing the error on [*Ag*]_0_.

### 3.4. Stochastic Variations in the Amount of Antibody

The uncertainty on the amount of added antibody influence $\frac{\left[B\right]}{\left[T\right]}$ not directly, since $\frac{\left[B\right]}{\left[T\right]}$ is independent of [*T*] in the absence of antigen depletion, but because the blank subtraction requires an equal [*T*] in all wells. The SD between the three matrices $\frac{\left[B\right]}{\left[T\right]}$ constructed for a 1% error on [*T*] are represented in a contour plot, Figure 3B.

The SDs for this error are in the range 0.003–0.010. The SDs are proportional to the unbound fraction (Equation (30)).

### 3.5. Stochastic Variations in the Nonspecific Binding

The well-to-well variations in nonspecific binding influence $\frac{\left[B\right]}{\left[T\right]}$ significantly. In a series of triplicate laboratory experiments (*n* = 40), the SD of the blank wells were measured on average at $\Delta U_{blank}=0.015\cdot U_{blank}$ (Figure S5). The resulting SD of $\frac{\left[B\right]}{\left[T\right]}$ can be approximated to $\sqrt{2}\cdot 0.015=0.021$ (Equation (38)). This error is constant.

### 3.6. Gamma Counter Errors

To account for the uncertainty caused by the counting of radioactivity, a constant $\Delta B=0.01\cdot U_{blank}$ is chosen, corresponding to a SD of $\frac{\left[B\right]}{\left[T\right]}$ equal to 0.01. This error threshold can usually be reached by selecting an appropriate counting time.

### 3.7. Overall Stochastic Errors

By applying a quadratic sum of errors (Equation (39)), the global stochastic error is computed. The overall SD of $\frac{\left[B\right]}{\left[T\right]}$ are illustrated in Figure 3C. Errors on the amount of antigen and in the nonspecific binding are the dominant elements of the sum. The overall SD are smoothed by the quadratic sum and relatively constant.

By dividing the overall SD matrix by the matrix of the original bound fractions, we obtain the matrix of coefficients of variation (Figure 3D). A high CV (10%–300%) is obtained when the denominator contains small bound fractions. These points correspond to *K_D_* > 3.16 nM and [*Ag*]_0_ < *K_D_*. The assumption of [*Ag*]_0_ ≫ *K_D_* that was not relevant for the ideal dataset is now important because stochastic variations become relatively large when not fulfilled. By using such randomly disturbed $\frac{\left[B\right]}{\left[T\right]}$ values, extrapolation errors of *r* and *K_D_* can occur, depending on the robustness of the fit.

### 3.8. Influence of Kinetics

**Figure 4:**
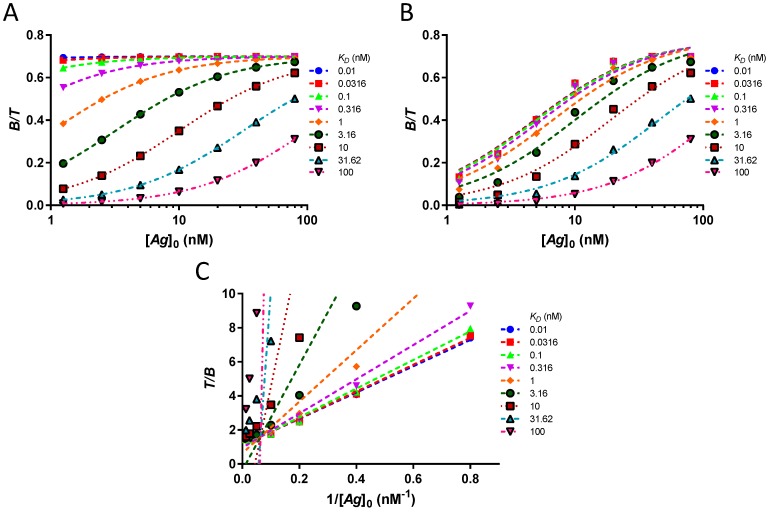
Influence of kinetics on fitted curves. (**A**) Theoretical bound fractions at equilibrium and approximate binding curves using hyperbola on a semi-logarithmic scale. (**B**) Modification of the dataset under conditions of nonattainment of equilibrium. The formation rates of the complex *B* depend on the antigen concentrations. Using a constant time *t* = 1200 min and *k_on_* = 0.0001444 nM^−1^min^−1^, the low bound fractions at [*Ag*]_0_ ≤ 5 nM are strongly reduced whilst the points at [*Ag*]_0_ ≥ 20 nM are close to the equilibrium values. When extrapolated with hyperbola, the binding curves overestimate the values of *K_D_* for the *K_D_* ≤ 3.16 nM. (**C**) Lindmo plots of the same dataset. The inversion of the ordinate amplifies the systematic error of kinetics: the value of the inverted data points increases rapidly as [*Ag*]_0_ decreases. For *K_D_* ≥ 1 nM, the low bound fractions at [*Ag*]_0_ ≤ 10 nM lead to large $\frac{\left[T\right]}{\left[B\right]}$ values that are no longer linearly distributed. The slope of the linear fits is increased and the extrapolated values of $\frac{1}{r}$ at *y*-intercept fall below 1.

**Figure 5:**
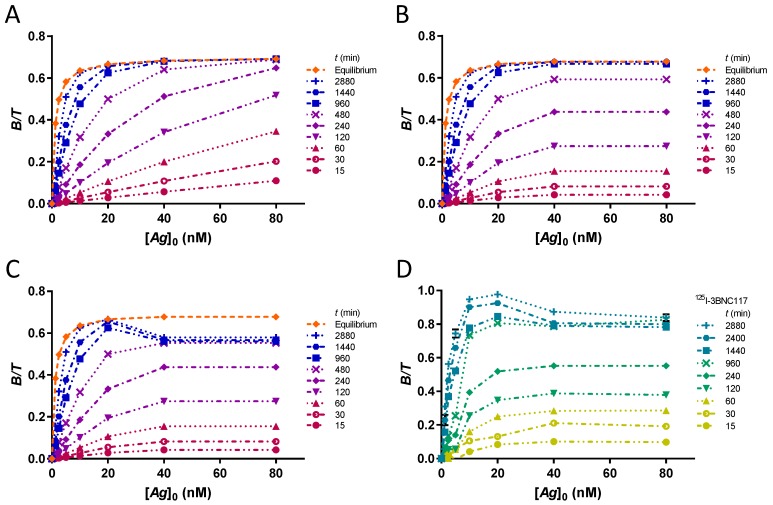
Time-course of formation of the *B* complex in kinetic assays. (**A**) Bound fractions at different times for a complex with *K_D_* = 1 nM and *k_on_* = 0.0001444 nM^−1^min^−1^ on an unsaturated plate. The formation rates of complex *B* depend on antigen concentrations. The theoretical binding curve at equilibrium is shown in orange. Data points are linked by curves for easy viewing. The curves show the growth over time while complex formation seeks to reach equilibrium values. (**B**) Bound fractions at different times for a saturated plate at [*Ag*]_0_ = 30 nM. The horizontal slope between the two highest antigen concentrations indicates similar growth rates and therefore, saturation. (**C**) Bound fractions at different times for a saturated plate at [*Ag*]_0_ = 30 nM, showing a time-dependent desorption. Bound fractions at the two highest [*Ag*]_0_ start regression from 480 min. The values $\frac{\left[B\right]}{\left[T\right]}$ finally reach a maximum at [*Ag*]_0_ = 20 nM. (**D**) Kinetic assay of the binding of the ^123^I-3BNC117 antibody to 426c core gp120 heptamer at 5 °C. In this laboratory experiment, we observed a time-dependent *B* complex desorption indicative of a saturated plate. The time required to reach 50% of the maximum bound fraction at [*Ag*]_0_ = 20 nM gave the value of the rate constant *k_on_* = 0.0001444 nM^−1^min^−1^ used to construct the simulation.

The systematic error of kinetics depends on [*Ag*]_0_ as follows. The dataset of theoretical binding curves at equilibrium (Figure 1A) is shown in a semi-logarithmic graph in Figure 4A. To model the unattained equilibrium, we multiply the original values by the integrated rate function for time *t* = 1200 min. The rate function ($1-e^{-k_{on}\cdot {\left[Ag\right]}_{0}\cdot t}$) requires a value for the *k_on_* parameter that has been obtained from a kinetic assay (Figure 5D). A 240 min half time of complex *B* formation was found at [*Ag*]_0_ = 20 nM. The corresponding rate constant *k_on_* is calculated:

(46) $$k_{on}=\frac{\ln2}{t_{\frac{1}{2}}\cdot {\left[Ag\right]}_{0}}=0.0001444\;nM^{-1}min^{-1}=2.4\cdot 10^{3}\;M^{-1}s^{-1}$$

The same *k_on_* is introduced at each [*Ag*]_0_, which causes different kinetics due to the different values of [*Ag*]_0_ (Figure 4B). Slow kinetics at low [*Ag*]_0_ systematically reduce the respective bound fractions, giving binding curves that mimic the curves obtained with higher *K_D_* values at equilibrium. The corresponding Lindmo plots are shown in Figure 4C. The linear relationship is lost, leading to impossible values for *r*.

In Table 2, the influence of kinetics on the estimates of *r* and *K_D_* is detailed for the complete dataset and for the dataset obtained after excluding values $\frac{\left[B\right]}{\left[T\right]}$ at levels of [*Ag*]_0_ ≤ 5 nM.

The data show that the extrapolation of *r* using hyperbola is robust and can be performed with or without exclusion of low fractions bound at the smaller [*Ag*]_0_, which are the most affected by kinetics. On the contrary, the fit using the Lindmo plot gives nonsensical values for *r*, unless the data points at the low levels [*Ag*]_0_ are excluded. None of these methods provide good estimates of *K_D_* values that are unduly overestimated.

### 3.9. Study of PS Saturation and B Desorption

The time-course of formation of the *B* complex provides important information for understanding IRFA. In particular, one can deduce the equilibration time for any [*Ag*]_0_. For instance, by comparing the rate constant $k_{on}^{\prime }$ for each [*Ag*]_0_, we can say with certainty—since the rate constant is proportional to [*Ag*]_0_—that the low bound fractions have not yet reached equilibrium (Figure 5A). Conversely, since [*Ag*]_0_ is included in the integrated rate function, a parallel growth of $\frac{\left[B\right]}{\left[T\right]}$ resulting from $k_{on}^{\prime }$ equal for multiple [*Ag*]_0_ implies that the PS is saturated with antigen (Figure 5B). Sometimes, the growth rate of $\frac{\left[B\right]}{\left[T\right]}$ for the highest levels of [*Ag*]_0_ decelerates and the growth of $\frac{\left[B\right]}{\left[T\right]}$ at a lower level of [*Ag*]_0_ supersedes it (Figure 5C). This is understood as a time-dependent desorption of complex *B*, which is recovered in the supernatant. As a result, the $\frac{\left[B\right]}{\left[T\right]}$ at the highest levels of [*Ag*]_0_ are artificially lowered, which may have an adverse effect on the ability to determine *r*. Such a case occurs in the kinetic assay with the anti-gp120 ^125^I-3BNC117 antibody shown in Figure 5D.

### 3.10. Time-Dependence of the Estimation of r

By observing these time-varying curves (Figure 5), it is necessary to determine when the estimation of *r* from the curves becomes significant, that is to say the robustness of the extrapolation of *r* for a shorter incubation time.

**Figure 6:**
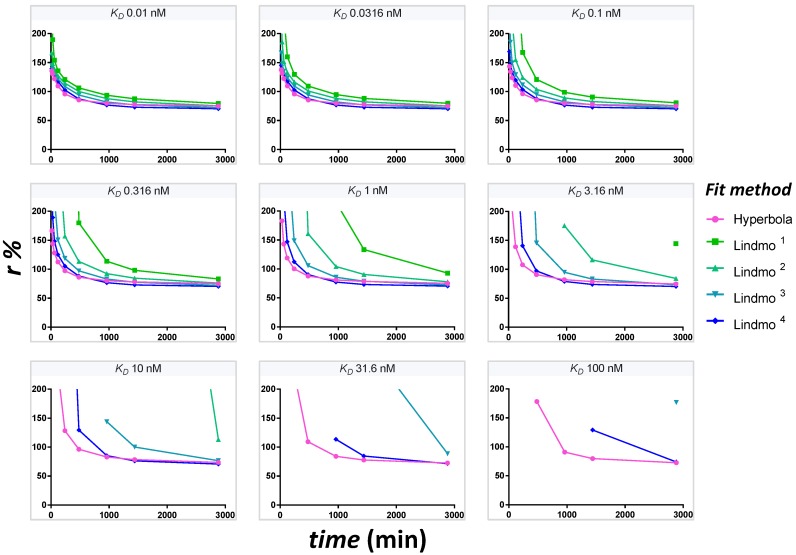
The hyperbola gives the best fit for r for all times and all *K_D_*. The estimates of *r* are extrapolated from the binding curve at different times for an unsaturated PS plate with [*T*] = 0.1 nM. There is no stochastic error. As the duration increases, the estimated values of *r* converge towards the expected value of *r* = 70%. Hyperbola (magenta curve) allows for faster extrapolation, increased accuracy and greater robustness. Lindmo plots (green-blue curves: ^1^ with all data points, ^2^ exclusion at [*Ag*]_0_ = 1.25 nM, ^3^ exclusion at [*Ag*]_0_ ≤ 2.5 nM, ^4^ exclusion at [*Ag*]_0_ ≤ 5 nM) approach the target *r* value at a slower pace, are less accurate (as shown by wide curves) and are not as robust (presence of several negative *r* values—points not represented). Although the exclusion of low bound fractions increases the accuracy of the Lindmo plot, hyperbola without any exclusion of data points is the best fit in case of nonequilibrium conditions, especially at higher *K_D_*.

The time-dependence of *r* estimates from binding curves that have not yet reached full dynamic equilibrium is detailed for nine *K_D_* values (Figure 6). Hyperbola and Lindmo plot with stepwise exclusion of data points at [*Ag*]_0_ ≤ 5 nM are compared. The hyperbola offers the best solution for the rapid determination of *r*, for increased resistance to non-achievement of equilibrium, for smaller fluctuations in values of *r* as a function of time and for a better accuracy.

In Figure S1 and S2, the time-dependence of *r* estimates in case of saturation and desorption is presented. The experimental data from the kinetic assay with ^125^I-3BNC117 are displayed in Figure S3 and S4.

### 3.11. Comparing Extrapolation Methods with Simulated Data

To compare the two fitting methods, namely the hyperbola and the Lindmo plot, a simulation is performed using the overall SD matrix and the dataset of bound fractions at a time *t* = 1200 min, containing several nonequilibrium data points. After the in silico generation of *n* = 25 matrices of pseudo-experimental data, the extrapolated *r* values are examined using mean and standard deviation (Table 3). The data demonstrate that the precision, accuracy, and robustness of estimates resulting from a fit with hyperbola are greater than those obtained using the Lindmo plot. The Lindmo plot shows several absurd values for *r* with considerable SD and requires the exclusion of the low bound fractions corresponding to ${\left[Ag\right]}_{0}=\left\{1.25;2.5;5\right\}\;\mathrm{nM}$ to give good estimates up to *K_D_* = 10 nM.

For *K_D_* estimates, the systematic kinetic error makes their values unreliable (Table S4). Another error related to *K*_D_ estimates occurs experimentally because not all applied antigens bind to the plate or have fully accessible binding sites. For these reasons, *K*_D_ should not be estimated from an IRFA. Similar results for *r* were obtained for simulated experiments showing saturation with or without desorption (Table S1 and S2).

### 3.12. Graphical Analysis of Simulated Data

**Figure 7:**
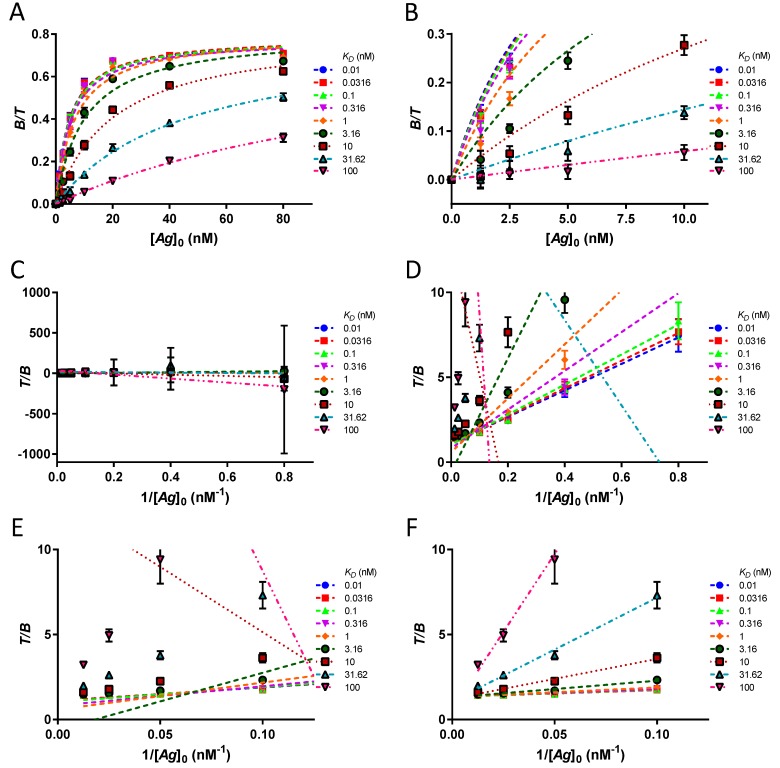
Compared to hyperbola, the Lindmo plot is chaotic, unless the low bound fractions are excluded. (**A**) Mean ± SD of bound fractions for ten experiments in triplicate and extrapolation of binding curves using hyperbola. (**B**) Detail of the previous graph at [*Ag*]_0_ ≤ 10 nM. The error bars are due to stochastic effects. The data points are depressed with respect to their hyperbolic fits because of kinetics. (**C**) Lindmo plot of the dataset, global view. The mathematical transformation overestimates the errors for the bound fractions at [*Ag*]_0_ ≤ 5 nM, leading to extremely large errors bars. (**D**) Lindmo plot, normal view. The chaotic behaviour of the fits for *K_D_* > 3.16 nM is observed. (**E**) Lindmo plot, detail. A zoom on the bound fractions at [*Ag*]_0_ ≥ 10 nM shows that only the fits for *K_D_* ≤ 0.316 nM are precise, but not accurate, when the whole dataset is considered. (**F**) When the bound fractions at [*Ag*]_0_ ≤ 5 nM are removed from the dataset, the goodness of fit becomes adequate for *K_D_* ≤ 10 nM.

The reasons for the poor performance of the Lindmo plot relative to hyperbola are disclosed in Figure 7A–F. A simulated dataset of *n* = 10 triplicates and their hyperbolic fits are illustrated in Figure 7A, B. The SD at the low $\frac{\left[B\right]}{\left[T\right]}$ and the unachieved equilibrium at these points do not radically alter the extrapolated hyperbola, as evidenced by the excellent goodness of fit. The corresponding Lindmo plots are presented in Figure 7C–F at different zoom levels. Here, mathematical distortions caused by inversion of the ordinate occur and artificially amplify the stochastic errors of $\frac{\left[B\right]}{\left[T\right]}$, in particular, at the three lowest [*Ag*]_0_ values (Figure 7C). In addition, owing to the unattained equilibrium, the slopes of the linear fits increase. The conjunction of the three effects—mathematical distortions, stochastic errors, and unreached equilibrium—completely mismatches the linear fits (Figure 7D–E). Only after the exclusion of the low bound fractions at [*Ag*]_0_ ≤ 5 nM will the Lindmo plots align with the data (Figure 7F).

## 4. Discussion

Despite the importance of the immunoreactive fraction assay (IRFA) for determining the purity of radioimmunoconjugates, there is no established method or guideline for quantifying functional quality. The true value of the immunoreactive fraction *r*, defined in dynamic equilibrium with an infinite excess of antigen, can only be approached by mathematical extrapolation. The most appropriate data analysis involves the direct representation of the bound fraction as a function of [*Ag*]_0_ in a conventional saturation binding curve shaped as a rectangular hyperbola (Langmuir isotherm) [23]. Historically, because of the difficulty for the human eye to correctly estimate a curve that extends to infinity, manual extrapolation of *r* with this method has hardly ever been used. In fact, in 1984, Lindmo et al. introduced a mathematical transformation to linearize the data through a double inversion, making the Lindmo plot the most common extrapolation method of *r* for its apparent simplicity [11].

In the past, some authors have recognized that the use of Lindmo plot may be problematic [8,9,12,16,22]. However, a systematic analysis of the individual problems and their effects on the extrapolation of *r* and *K_D_* was lacking. In the absence of consensus, the subject is made more difficult by the inability to compare extrapolated *r* values with a benchmark *r* value. Therefore, without a clear demonstration of its lack of accuracy, precision, and robustness, the Lindmo plot continues to be widely used.

Mathematically, the Lindmo plot is sound as it is easily derived from the hyperbola. With ideal data points at equilibrium, in the absence of antigen depletion, there is excellent goodness of fit of both approximate equations with the theoretical data. The extrapolated values of *r* and *K_D_* are correctly determined.

At first glance, the range is wide for the values of [*Ag*]_0_ and *K_D_*, the only assumption required for the ideal dataset being the antigen always in excess of the antibody. For instance, [*Ag*]_0_ ≫ *K_D_* is not necessary because the hyperbola and the linear fit of Lindmo plot are entirely determined by knowing only the exact bound fractions, regardless of their size. Moreover, the assumption [*T*] ≪ *K_D_* is not relevant if [*Ag*]_0_ ≫ [*T*]. If [*Ag*]_0_ ≫ [*T*] is not true for at least one [*Ag*]_0_, the adverse case of antigen depletion is encountered. Then, errors are observed and become significant if the antigen is exhausted for several [*Ag*]_0_. A comparison of the Lindmo plot with hyperbola establishes that the Lindmo plot always provides worse estimates of *r* and *K_D_* than the hyperbola in the case of antigen depletion. However, this can be avoided simply by using a lower [*T*], if necessary, with a higher specific activity.

Unlike ideal data points, experimental data points are generally subject to uncertainties and incomplete equilibration. Since the two approximate equations give excellent estimates for the ideal dataset, but not in an experimental setting, we deduce that the problems attributed to the Lindmo plot result from mismanagement of the discrepancies between the ideal points and the experimental data. It is also interesting to know whether the mathematical transformation of Lindmo plot exacerbates these errors or whether hyperbola is also subject to them. To challenge the accuracy, precision, and robustness of the Lindmo plot and hyperbola, an in silico simulation was performed. In this way, the estimates of *r* and *K_D_* obtained with the two approximate equations from the simulated datasets can be directly compared to the original input values.

Three types of errors were examined: stochastic, kinetic and PS saturation. To model the stochastic variations in the pseudo-experimental data, standard deviations matrices were computed. The two dominant elements of the quadratic sum of SD are the uncertainties on [*Ag*]_0_ and on the nonspecific binding. SD are relatively constant over the entire range of *K_D_* and [*Ag*]_0_. Nevertheless, CV show a wide range (3–300%). This contrasts with Lindmo et al. which assumes a constant CV value [11]. Owing to the minimized CV at [*Ag*]_0_ > *K_D_*, the assumption of [*Ag*]_0_ ≫ *K_D_* is now gaining importance with the use of a disturbed dataset in order to avoid errors in the estimates of *r* and *K_D_*.

The error of kinetics was investigated using a mono-exponential rate law reaching the equilibrium values at each [*Ag*]_0_. In IRFA, the kinetics depend on [*Ag*]_0_ and not on the radioligand concentration. Indeed, when [*Ag*]_0_ ≫ [*T*] the association reaction follows a pseudo-first order rate which includes [*Ag*]_0_ in the rate constant. With decreasing [*Ag*]_0_, at some point, the rate constant is small enough that the equilibrium is not reached within a reasonable time, despite resembling a steady state because of slow association kinetics. This leads to a systematic depression of the bound fractions at the low [*Ag*]_0_. A dataset including points that are not in equilibrium leads to extrapolation errors of *r* and *K_D_*. With hyperbola, a stable overestimation of *r* of about 10% is obtained. The *K_D_* values are overestimated. The same dataset extrapolated with the Lindmo plot give estimates of *r* > 100%, even negative. Thus, the Lindmo plot, but not the hyperbola, requires the exclusion of data points at low [*Ag*]_0_ in case of incomplete equilibration. Similar results were obtained by Hulme in saturation assays of antigen (Scatchard) [20]. However, in IRFA (saturation assays of antibody), this kinetic effect is more problematic. In Scatchard, the kinetics depend on the excess concentration of radioligand in solution and not on coated [*Ag*]_0_ as in IRFA. A lower [*Ag*]_0_ can be coated on a solid surface than the radioligand concentration available in Scatchard, thus slowing kinetics. Complexes with low *k_on_* aggravate this slowdown. In this study, the complex has a low *k_on_* value ($2.4\cdot 10^{3}\;M^{-1}s^{-1}$). However, we used a long incubation period of 20 h. High affinity or avidity and the low temperature of 5 °C used in our laboratory experiments may explain the measurement of a low *k_on_* [20,31]. Complexes with such a low *k_on_* have been reported [12,32]. With the trend of developing long residence time and potent ligands, the problem of slow kinetics in IRFA will probably remain [33,34]. The influence of incomplete equilibration is scarcely recognized because discrepancies revealed in the data are imputed in place to insufficient antigen excess and antigen depletion [8,9,11,22].

The saturation of the PS is another source of systematic error. The time-course of complex formation is useful for establishing the equilibrium time at different [*Ag*]_0_. Owing to the presence of [*Ag*]_0_ in the rate constant, the equilibration time for each [*Ag*]_0_ can be compared. By observing equal equilibration times for different [*Ag*]_0_, one can deduce the saturation of the PS plate in antigen. Thus, crucial information for understanding the IRFA is obtained from kinetic assays.

Using several equilibration times, the time-dependence of the estimation of *r* was investigated. We showed that a fit using a hyperbola always provides a more accurate extrapolation of *r* at an earlier time, with greater precision and robustness than a Lindmo plot. The Lindmo plot requires the exclusion of the low bound fractions to provide better estimates of *r*.

By combining the ideal dataset, the kinetic function and the standard deviations matrix, the final simulation was performed. The pseudo-random dataset provides disturbed bound fractions and unattained equilibrium that model an experimental setting. The estimation of *r* using the hyperbola is precise and robust, but slightly inaccurate because of kinetics. The Lindmo plot is fragile and chaotic owing to the artificial amplification of fluctuations at the low bound fractions. The causal factors affecting the Lindmo plot were disclosed in a graphical analysis. Depending on the binding isotherm, the bound fractions at low [*Ag*]_0_ are low. At these low [*Ag*]_0_ values, additional depression occurs because of slower kinetics. SD being relatively constant, significant CV appear. Inversion of the ordinate with the Lindmo plot creates mathematical distortions resulting from poor error management. This changes the scale of stochastic variations from small absolute values to extremely large random fluctuations. A linear fit with the complete dataset generates nonsensical *r* values, unless the low bound fractions are excluded.

## 5. Conclusions

The Lindmo plot is the most common method for determining the immunoreactive fraction of radiolabeled proteins and antibodies. It employs linear extrapolation of a plot of saturation binding data in antigen excess. However, significant errors in estimates of the functional quality of radiotracers, particularly those with slow kinetics such as radiolabeled antibodies, are obtained by this method. Using simulated and experimental data, we proved that the fit with the rectangular hyperbola is superior—in accuracy, precision, and robustness—compared to the Lindmo plot for the extrapolation of *r*. In this study, the experimental data consist of saturation binding assays with coated antigens. Cell-based assays were not used but, even so, the conclusion remains the same. Indeed, the simulation of several binding datasets mathematically shows that the extrapolation of binding data using the double inverse Lindmo plot is the cause of errors when the data at the low bound fractions are not excluded, because of poor error management. With modern data analysis software that allows non-linear regression of the dataset, the Lindmo plot is obsolete. The saturation isotherm of immunoreactive fraction assays is advantageously extrapolated with a rectangular hyperbola, which we now recommend to use. We believe that this method will be useful in the future clinical translation of radiolabeled proteins and antibodies for imaging and theranostic applications. 

## Figures and Tables

**Table 1:** Individual certainties for each error considered.

Certainties	Stochastic Variations				Systematic Errors	
	[*Ag*]_0_	*T*	B_NS_	γ_count_	Kin ^1^	PS ^2^
[*Ag*]_0_ exact	✘	✔	✔	✔	✔	✔
*T* constant	✔	✘	✔	✔	✔	✔
No *B_NS_*	✔	✔	✘	✔	✔	✔
γ_count_ exact	✔	✔	✔	✘	✔	✔
At equilibrium	✔	✔	✔	✔	✘	✘
PS not saturated	✔	✔	✔	✔	✔	✘
*U* exact ^3^	✔	✔	✔	✔	✔	✔

^1^ Equilibrium is not attained; ^2^ PS is saturated; ^3^ *U* is exactly quantified (there is no volumetric error on *U*). Legend: ✔ = true, ✘ = false.

**Table 2:** The Lindmo plot is more affected by kinetics than hyperbola.

Fit Method	*K_D_* (nM)	0.01	0.0316	0.1	0.316	1	3.16	10	31.62	100
Hyperbola ^3^	*r* (%) ^1^	78	79	79	79	80	80	80	80	84
	*r* (%) ^2^	74	74	74	74	74	74	75	75	78
	*K_D_* ^1^	4.64	4.69	4.85	5.33	6.64	10.0	19.0	45.3	133
	*K_D_* ^2^	2.53	2.56	2.64	2.91	3.74	6.35	14.3	39.0	119
Lindmo ^4^	*r* (%) ^1^	90	91	94	104	158	*	*	*	*
	*r* (%) ^2^	74	74	74	74	75	76	80	94	216
	*K_D_* ^1^	6.95	7.16	7.88	10.5	24.0	*	*	*	*
	*K_D_* ^2^	2.73	2.76	2.85	3.15	4.08	7.10	17.2	56.7	396

Results of fits with total antibody [*T*] = 0.1 nM, *r* = 70%, at *t* = 1200 min and *k_on_* = 0.0001444 nM^−1^min^−1^. * negative value; ^1^ the antigen concentrations ${\left[Ag\right]}_{0}=\left\{1.25;2.5;5;10;20;40;80\right\}\;\mathrm{nM}$ are used; ^2^ the antigen concentrations ${\left[Ag\right]}_{0}=\left\{10;20;40;80\right\}\;\mathrm{nM}$ are used; ^3^ with hyperbola, the inability to reach equilibrium induces a systematic error of about 10% of *r*. *K_D_* estimates are incorrectly high. When the bound fractions at [*Ag*]_0_ ≤ 5 nM are excluded, the values of *r* and *K_D_* change marginally; ^4^ Lindmo plot gives overestimated *r* values. Nonsense *r* estimates are obtained for *K_D_* ≥ 1 nM. *K_D_* estimates are incorrectly high and absurd (negative) for *K_D_* > 1 nM. When excluding the bound fractions at [*Ag*]_0_ ≤ 5 nM, the accuracy of the *r* values increases and reflects the *r* values obtained with the hyperbola, except at *K_D_* > 10 nM.

**Table 3:** Lindmo plot shows significant extrapolation errors unless low bound fractions are excluded.

Fit Method	*K_D_* (nM)	0.01	0.0316	0.1	0.316	1	3.16	10	31.62	100
Hyperbola ^5^	*r* (%) ^1^	78 ± 1	78 ± 1	79 ± 1	79 ± 1	79 ± 1	80 ± 1	80 ± 2	81 ± 4	104 ± 46
	*r* (%) ^2^	78 ± 1	78 ± 1	78 ± 1	78 ± 1	78 ± 1	79 ± 1	80 ± 2	80 ± 4	103 ± 46
	*r* (%) ^3^	76 ± 1	76 ± 1	76 ± 1	77 ± 1	76 ± 2	77 ± 1	78 ± 2	78 ± 4	101 ± 44
	*r* (%) ^4^	74 ± 1	73 ± 1	74 ± 2	74 ± 1	74 ± 1	74 ± 2	75 ± 2	76 ± 4	97 ± 42
Lindmo ^6^	*r* (%) ^1^	98 ± 19	93 ± 15	99 ± 16	102 ± 19	127 ± 246	55 ± 645	31 ± 123	15 ± 42	0 ± 8
	*r* (%) ^2^	84 ± 4	85 ± 4	86 ± 4	90 ± 5	97 ± 11	116 ± 233	183 ± 980	*	6 ± 39
	*r* (%) ^3^	79 ± 2	79 ± 2	79 ± 2	80 ± 2	81 ± 4	88 ± 7	137 ± 79	*	22 ± 79
	*r* (%) ^4^	74 ± 1	74 ± 2	74 ± 2	74 ± 1	75 ± 2	77 ± 3	81 ± 6	127 ± 91	11 ± 183

Mean ± SD results of fits. The simulation uses *n* = 25 matrices of triplicate $\frac{[\tilde{B]}}{\left[T\right]}$ values with [*T*] = 0.1 nM, *r* = 70%, at *t* = 1200 min and *k_on_* = 0.0001444 nM^−1^min^−1^. * negative value; ^1^ the antigen concentrations ${\left[Ag\right]}_{0}=\left\{1.25;2.5;5;10;20;40;80\right\}\;\mathrm{nM}$ are used; ^2^ the antigen concentrations ${\left[Ag\right]}_{0}=\left\{2.5;5;10;20;40;80\right\}\;\mathrm{nM}$ are used; ^3^ the antigen concentrations ${\left[Ag\right]}_{0}=\left\{5;10;20;40;80\right\}\;\mathrm{nM}$ are used; ^4^ the antigen concentrations ${\left[Ag\right]}_{0}=\left\{10;20;40;80\right\}\;\mathrm{nM}$ are used; ^5^ with hyperbola, failure to achieve equilibrium induces a systematic error of about 10% of *r*. SD are small for *K_D_* ≤ 31.62 nM, indicating good precision. The exclusion of the bound fractions at [*Ag*]_0_ ≤ 5 nM does not modify the SD, but slightly decreases the systematic error of kinetics; ^6^ The Lindmo plot gives overestimated *r* values. Nonsense *r* estimates are obtained for *K_D_* ≥ 1 nM. The SD are substantial when the bound fractions at [*Ag*]_0_ ≤ 5 nM are not excluded. The gradual exclusion of these data points provides more and more precision and accuracy. After excluding the bound fractions at [*Ag*]_0_ ≤ 5 nM, the Lindmo plot gives good *r* estimates for *K_D_* < 10 nM.

## Supplementary Materials

The supplementary materials are available online at https://www.mdpi.com/1424-8247/12/4/177/s1. Figure S1. Time-dependence of the estimation of *r* in case of PS saturation. The values of *r* are extrapolated from the binding curve at different time points for a PS plate saturated at the level [*Ag*]_0_ = 30 nM. Hyperbola (magenta curve) allows for faster extrapolation, increased accuracy and greater robustness than Lindmo plots (green-blue curves: ^1^ with all data points, ^2^ exclusion at [*Ag*]_0_ = 1.25 nM, ^3^ exclusion at [*Ag*]_0_ ≤ 2.5 nM, ^4^ exclusion at [*Ag*]_0_ ≤ 5 nM). Negative *r* values (many occur in Lindmo plots) are not shown. The Lindmo plots give bad fit, requiring the exclusion of the low bound fractions. Hyperbola, which does not require exclusion of data points, is the most reliable fit. However, for a saturated plate, the accuracy of *r* at *K_D_* > 10 nM decreases even with hyperbola. Figure S2. Time-dependence of the estimation of *r* in case of desorption of *B*. The values of *r* are extrapolated from the binding curve at different time points for a PS plate saturated at the level [*Ag*]_0_ = 30 nM and showing a desorption of 10% of *B*. These curves are very similar to those without desorption (Figure S1). Hyperbola (magenta curve) allows faster extrapolation, higher accuracy, and greater robustness than Lindmo plots (green-blue curves: ^1^ with all data points, ^2^ exclusion at [*Ag*]_0_ = 1.25 nM, ^3^ exclusion at [*Ag*]_0_ ≤ 2.5 nM, ^4^ exclusion at [*Ag*]_0_ ≤ 5 nM). Negative *r* values (not shown) occur with Lindmo plots but not with hyperbola. The Lindmo plot requires exclusion of the low bound fractions, but not hyperbola. The accuracy of *r* at *K_D_* > 10 nM is poor, which limits the assay to *K_D_* ≤ 10 nM in case of a PS plate saturated at this level of [*Ag*]_0_. Figure S3. Time-dependence of the estimation of *r* for anti-gp120 ^125^I-3BNC117 antibody bound to an excess of gp120 heptamer. The values of *r* are extrapolated from the binding curve at different incubation times. The hyperbola (magenta curve) provides a faster extrapolation, more precision and a higher robustness than the Lindmo plots (green-blue curves: ^1^ with all data points, ^2^ exclusion at [*Ag*]_0_ = 1.25 nM, ^3^ exclusion at [*Ag*]_0_ ≤ 2.5 nM, ^4^ exclusion at [*Ag*]_0_ ≤ 5 nM). Negative *r* values (not shown) occur with Lindmo plots but not with hyperbola. The Lindmo plot following exclusion of [*Ag*]_0_ ≤ 5 nM displays a level of precision and robustness similar to that of the hyperbola, with a lower *r* estimate. Figure S4. Approximate binding curves at each time point of the kinetic assay of anti-gp120 ^125^I-3BNC117 antibody bound to an excess of gp120 heptamer. In conditions of non-achievement of equilibrium, the bound fractions obtained are fitted with the approximate equations of hyperbola (A) and Lindmo plot (B). (A) This laboratory experiment shows saturation at [*Ag*]_0_ ≥ 40 nM and time-dependent desorption of the saturated data points after a time *t* ≥ 960 min. Extrapolation with hyperbola corrects some of these artifacts. (B) For the Lindmo plot, data at [*Ag*]_0_ ≤ 5 nM are excluded, which reduces the impact of the non-achievement of the equilibrium on the goodness of fit. Figure S5. Scatter plot of the measured $\Delta U_{blank}$ from *n* = 40 laboratory experiments in triplicate. The $\Delta U_{blank}$ of the blank wells were measured on average at $\Delta U_{blank}=0.015\cdot U_{blank}$. Table S1. Estimates of *r* extrapolated from *n* = 25 matrices of simulated experiments in triplicate for a saturated PS plate at [*Ag*]_0_ = 30 nM. Table S2. Estimates of *r* extrapolated from *n* = 25 matrices of simulated experiments in triplicate for a saturated plate showing desorption of *B*. Table S3. The approximate equations 10 and 11 provide excellent estimates of *r* and *K_D_* with the theoretical dataset at equilibrium and in the absence of antigen depletion. Table S4. Extrapolated *K_D_* values from *n* = 25 matrices of simulated experiments in triplicate.
