# Supplementary material for: A Robust Method for Assaying the Immunoreactive Fraction in Nonequilibrium Systems
Source: Pharmaceuticals (Basel). 2019 Dec 3;12(4):177. doi: 10.3390/ph12040177 (PMC6958493; doi:10.3390/ph12040177)
Supplement: Supplementary file 1 [file pharmaceuticals-12-00177-s001.pdf]

Supplementary information

A Robust Method for Assaying the Immunoreactive Fraction in Nonequilibrium Systems

Thibaut Denoël [1,*], Luca Pedrelli [1], Giuseppe Pantaleo [2] and John O. Prior [1]

[1] Department of Nuclear Medicine and Molecular Imaging, Lausanne University Hospital and University of Lausanne, Rue du Bugnon 46, 1011 Lausanne (Switzerland); thibaut.denoel@chuv.ch (T.D.); luca.pedrelli@alumni.epfl.ch (L.P.); john.prior@chuv.ch (J.O.P.)

[2] Division of Immunology and Allergy, Department of Medicine, Lausanne University Hospital and University of Lausanne, Rue du Bugnon 46, 1011 Lausanne (Switzerland); giuseppe.pantaleo@chuv.ch (G.P.)

* Correspondence: thibaut.denoel@chuv.ch; Tel.: +41-021-3144326

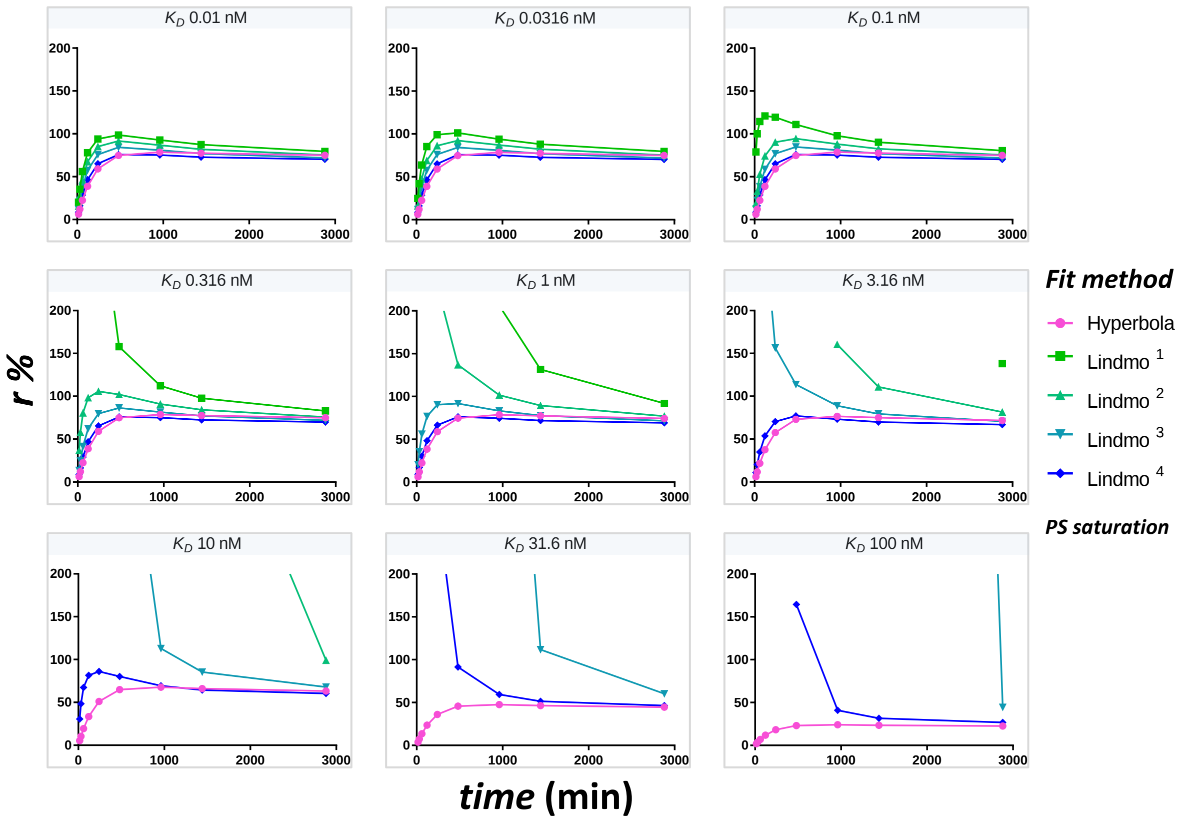

Figure S1. Time-dependence of the estimation of r in case of PS saturation. The values of r are extrapolated from the binding curve at different time points for a PS plate saturated at the level $[Ag]_0$ = 30 nM. Hyperbola (magenta curve) allows for faster extrapolation, increased accuracy and greater robustness than Lindmo plots (green-blue curves: [1] with all data points, [2] exclusion at $[Ag]_0$ = 1.25 nM, [3] exclusion at $[Ag]_0 \leq$ 2.5 nM, [4] exclusion at $[Ag]_0 \leq$ 5 nM). Negative r values (many occur in Lindmo plots) are not shown. The Lindmo plots give bad fit, requiring the exclusion of the low bound fractions. Hyperbola, which does not require exclusion of data points, is the most reliable fit. However, for a saturated plate, the accuracy of r at K_D > 10 nM decreases even with hyperbola.

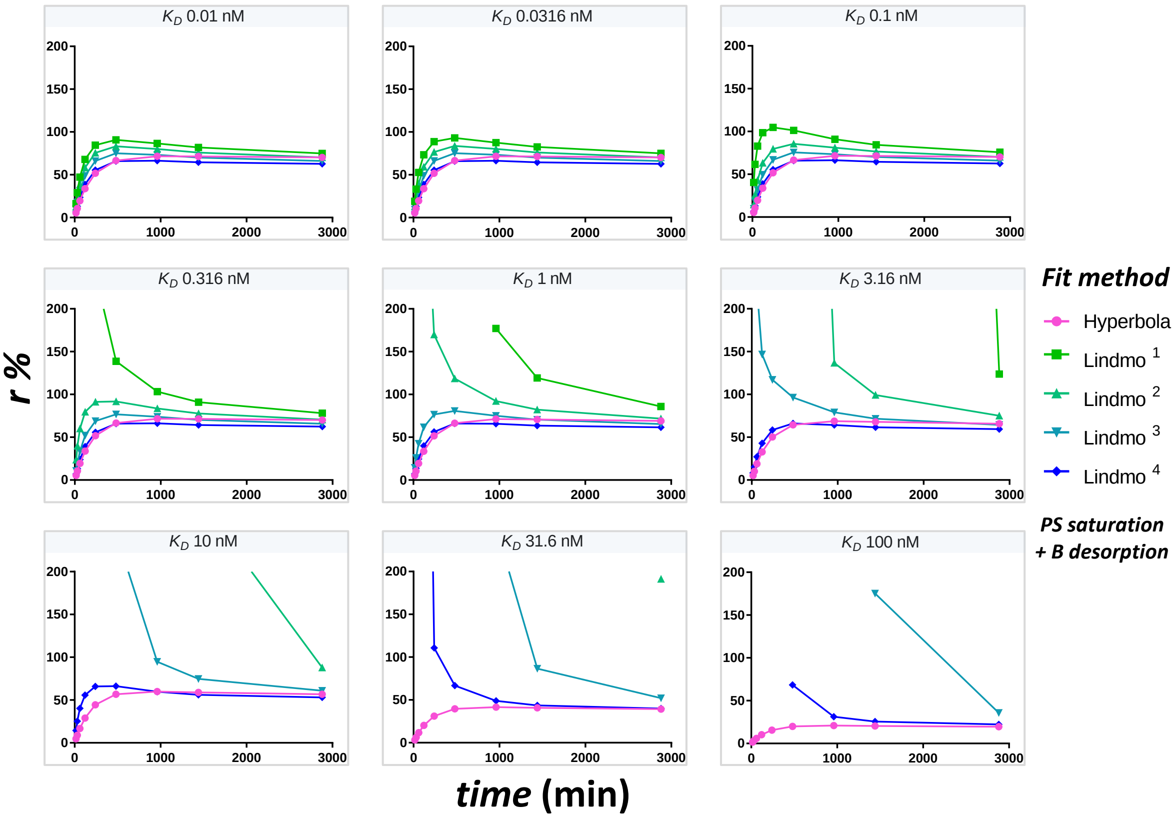

Figure S2. Time-dependence of the estimation of r in case of desorption of B. The values of r are extrapolated from the binding curve at different time points for a PS plate saturated at the level $[Ag]_0$ = 30 nM and showing a desorption of 10% of B. These curves are very similar to those without desorption (Figure S1). Hyperbola (magenta curve) allows faster extrapolation, higher accuracy, and greater robustness than Lindmo plots (green-blue curves: [1] with all data points, [2] exclusion at $[Ag]_0$ = 1.25 nM, [3] exclusion at $[Ag]_0 \leq 2.5$ nM, [4] exclusion at $[Ag]_0 \leq 5$ nM). Negative r values (not shown) occur with Lindmo plots but not with hyperbola. The Lindmo plot requires exclusion of the low bound fractions, but not hyperbola. The accuracy of r at $K_D > 10$ nM is poor, which limits the assay to $K_D \leq 10$ nM in case of a PS plate saturated at this level of $[Ag]_0$.

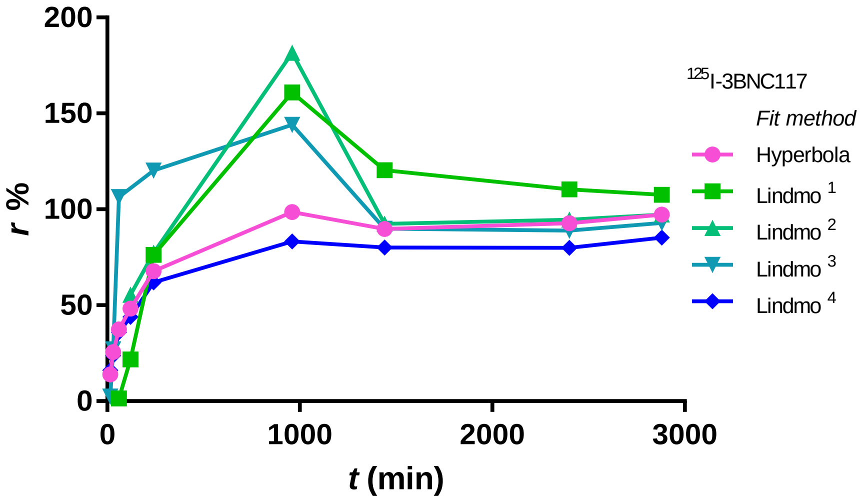

Figure S3. Time-dependence of the estimation of *r* for anti-gp120 ^{125}I-3BNC117 antibody bound to an excess of gp120 heptamer. The values of *r* are extrapolated from the binding curve at different incubation times. The hyperbola (magenta curve) provides a faster extrapolation, more precision and a higher robustness than the Lindmo plots (green-blue curves: [1] with all data points, [2] exclusion at $[Ag]_0$ = 1.25 nM, [3] exclusion at $[Ag]_0 \leq 2.5$ nM, [4] exclusion at $[Ag]_0 \leq 5$ nM). Negative *r* values (not shown) occur with Lindmo plots but not with hyperbola. The Lindmo plot following exclusion of $[Ag]_0 \leq 5$ nM displays a level of precision and robustness similar to that of the hyperbola, with a lower *r* estimate.

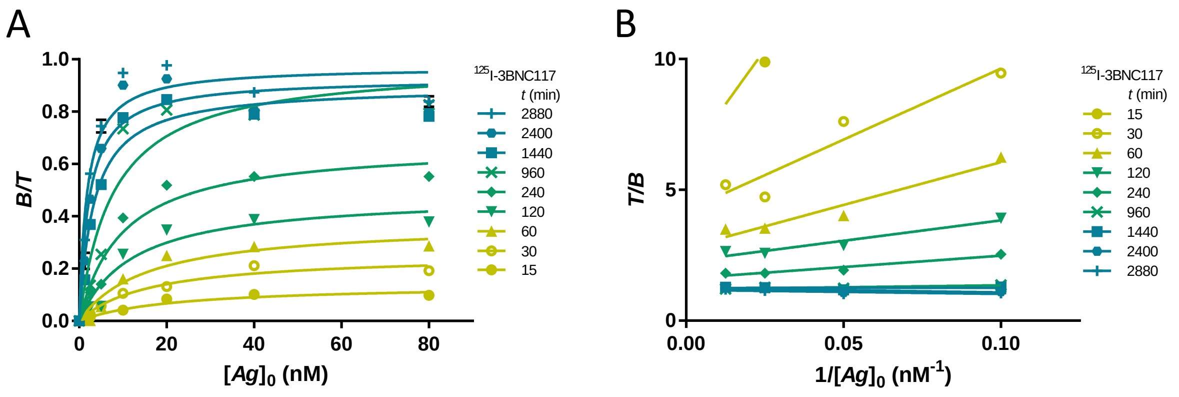

Figure S4. Approximate binding curves at each time point of the kinetic assay of anti-gp120 ^{125}I-3BNC117 antibody bound to an excess of gp120 heptamer. In conditions of non-achievement of equilibrium, the bound fractions obtained are fitted with the approximate equations of hyperbola (A) and Lindmo plot (B). (A) This laboratory experiment shows saturation at $[Ag]_0 \geq 40$ nM and time-dependent desorption of the saturated data points after a time $t \geq 960$ min. Extrapolation with hyperbola corrects some of these artifacts. (B) For the Lindmo plot, data at $[Ag]_0 \leq 5$ nM are excluded, which reduces the impact of the non-achievement of the equilibrium on the goodness of fit.

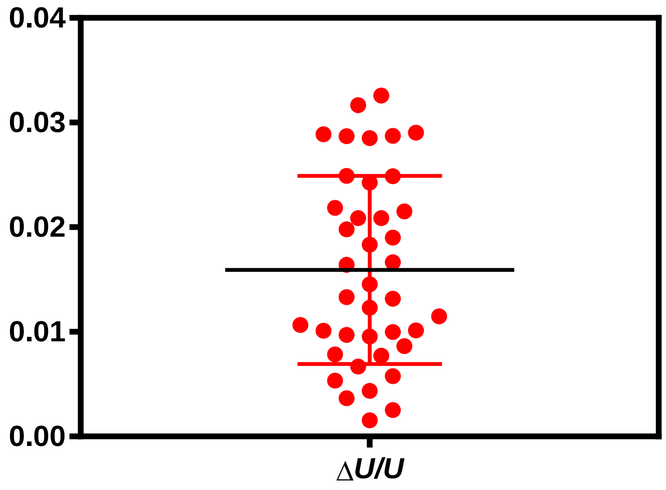

Figure S5. Scatter plot of the measured ΔU_{blank} from n = 40 laboratory experiments in triplicate. The ΔU_{blank} of the blank wells were measured on average at $\Delta U_{blank} = 0.015 \cdot U_{blank}$.

Table S1. Estimates of r extrapolated from n = 25 matrices of simulated experiments in triplicate for a saturated PS plate at $[Ag]_0$ = 30 nM.

Fit method	K_D (nM)	0.01	0.0316	0.1	0.316	1	3.16	10	31.62	100
Hyperbola	r (%) [1]	79±2	79±1	79±1	79±1	79±2	77±1	68±2	48±3	23±3
	r (%) [2]	83±2	83±2	84±2	84±2	85±2	87±3	85±5	77±20	58±46
Lindmo	r (%) [3]	75±2	75±2	74±2	74±1	73±2	73±2	68±4	60±13	28±48
	r (%) [4]	77±3	77±2	78±3	77±3	77±3	79±4	81±7	85±142	121±251

Mean±SD results of fits. The simulation uses n = 25 matrices of triplicate $\frac{[\widehat{B}]}{[T]}$ values, with $[T]$ = 0.1 nM, r = 70%, at t = 1200 min and k_{on} = 0.0001444 $nM^{-1}min^{-1}$. [1] the antigen concentrations $[Ag]_0 = \{1.25; 2.5; 5; 10; 20; 30; 30\}$ nM are used; [2] the antigen concentrations $[Ag]_0 = \{1.25; 2.5; 5; 10; 20; 30\}$ nM are used; [3] the antigen concentrations $[Ag]_0 = \{10; 20; 30; 30\}$ nM are used; [4] the antigen concentrations $[Ag]_0 = \{10; 20; 30\}$ nM are used. *First row:* if a kinetic assay has not been performed, it is not known if there is saturation and duplicated wells at $[Ag]_0$ = 30 nM. Therefore, extrapolation with hyperbola is done with the entire vector of antigen in the usual manner. Precision is good with small SD (1–3), but accuracy at K_D > 10 nM decreases. *Second row:* after acknowledging saturation, the duplicated well can be removed from the fit. In fact, this results in a poorer fit with decreased accuracy at $K_D \leq 10$ nM and an increase in SD. *Third row:* the Lindmo plot following the exclusion of the fractions bound at $[Ag]_0 \leq 5$ nM, but keeping the duplicate at $[Ag]_0$ = 30 nM, gives good estimates of r for $K_D \leq 10$ nM. *Fourth row:* after removing the duplicate at $[Ag]_0$ = 30 nM, using only three out of seven data points, precision and accuracy decrease.

Table S2. Estimates of r extrapolated from n = 25 matrices of simulated experiments in triplicate for a saturated plate showing desorption of B.

Fit method	K_D (nM)	0.01	0.0316	0.1	0.316	1	3.16	10	31.62	100
Hyperbola	r (%) [1,5]	72±1	72±1	72±1	72±1	71±1	69±1	60±2	41±2	21±3
	r (%) [2,5]	77±2	78±1	77±1	78±2	78±2	78±2	73±3	58±8	45±31
Lindmo	r (%) [3,5]	66±1	66±1	66±1	66±2	65±2	64±2	59±3	45±5	40±107
	r (%) [4,5]	72±15	72±16	71±13	72±14	71±16	72±16	72±14	68±25	41±79

Mean±SD results of fits. The simulation uses n = 25 matrices of triplicate $\frac{[\widehat{B}]}{[T]}$ values, with $[T]$ = 0.1 nM, r = 70%, at t = 1200 min and k_{on} = 0.0001444 $nM^{-1}min^{-1}$. [1] the antigen concentrations $[Ag]_0 = \{1.25; 2.5; 5; 10; 20; 30; 30\}$ nM are used; [2] the antigen concentrations $[Ag]_0 = \{1.25; 2.5; 5; 10; 20; 30\}$ nM are used; [3] the antigen concentrations $[Ag]_0 = \{10; 20; 30; 30\}$ nM are used; [4] the antigen concentrations $[Ag]_0 = \{10; 20; 30\}$ nM are used; [5] a multiplier of 0.9 was applied on the $\frac{[\widehat{B}]}{[T]}$ at $[Ag]_0 = 30$ nM to simulate 10% B desorption. *First row:* using the entire antigen vector, precision and accuracy are excellent up to $K_D \geq 10$ nM, at which point the accuracy of the r estimates decreases. *Second row:* upon removal of the duplicated well, the precision remains good for the estimation of r up to $K_D \geq 31.62$ nM. *Third row:* the Lindmo plot after excluding the fractions bound at $[Ag]_0 \leq 5$ nM, but keeping the duplicate at $[Ag]_0$ = 30 nM, gives precise but slightly depressed results up to $K_D \geq 10$ nM, which leads to degraded estimates. *Fourth row:* after removing the duplicate at $[Ag]_0$ = 30 nM, using only three out of seven data points, the precision deteriorates considerably.

Table S3. The approximate equations 10 and 11 provide excellent estimates of *r* and K_D with the theoretical dataset at equilibrium and in the absence of antigen depletion.

**Fit method**	**K_D (nM)**	**0.01**	**0.0316**	**0.1**	**0.316**	**1**	**3.16**	**10**	**31.62**	**100**
Hyperbola	*r* (%)	70.0	70.0	70.0	70.1	70.1	70.1	70.1	70.0	70.0
	K_D	0.011	0.033	0.105	0.330	1.028	3.204	10.05	31.69	100.1
Lindmo	*r* (%)	70.0	70.0	70.0	70.1	70.1	70.2	70.1	70.1	70.0
	K_D	0.011	0.033	0.105	0.331	1.034	3.222	10.08	31.72	100.1

Results of fits using equations 10 (hyperbola) and 11 (Lindmo plot). The theoretical $\frac{[B]}{[T]}$ values at equilibrium (equation 16), with [T] = 0.1 nM, r = 70% and $[Ag]_0 = \{1.25; 2.5; 5; 10; 20; 40; 80\}$ nM are used.

Table S4. Extrapolated K_D values from $n = 25$ matrices of simulated experiments in triplicate.

**Fit method**	**K_D (nM)**	**0.01**	**0.0316**	**0.1**	**0.316**	**1**	**3.16**	**10**	**31.62**	**100**
Hyperbola [5]	K_D [1]	4.7±0.3	4.6±0.3	4.9±0.3	5.4±0.4	6.6±0.4	10.1±0.6	19±1.7	45±5	178±101
	K_D [2]	4.4±0.3	4.4±0.3	4.5±0.3	5.1±0.4	6.0±0.4	9.5±0.7	18.4±1.7	44±5	175±100
	K_D [3]	3.8±0.3	3.7±0.3	3.9±0.4	4.3±0.5	5.1±0.5	8.3±0.7	16.9±1.7	43±5	172±97
	K_D [4]	2.6±0.4	2.5±0.6	2.6±0.5	2.9±0.5	3.7±0.6	6.4±1.0	14.3±1.6	39±5	160±92
Lindmo [6]	K_D [1]	8.6±3.6	7.6±2.9	8.9±2.8	10.3±3.7	17±50	5±174	9±69	8±44	*
	K_D [2]	6.0±1	6.1±0.9	6.3±0.9	7.9±1.3	11.1±3	22±72	69±440	*	5±82
	K_D [3]	4.5±0.5	4.5±0.5	4.7±0.7	5.4±0.9	6.6±1.2	12.3±2.7	48±43	*	17±157
	K_D [4]	2.8±0.6	2.7±0.7	2.8±0.6	3.1±0.5	4.1±0.7	7.4±1.4	18±4	90±92	*

Mean±SD results of fits. The simulation uses $n = 25$ matrices of triplicate values $\frac{[\widetilde{B}]}{[T]}$, with $[T]$ = 0.1 nM, r = 70%, at t = 1200 min and k_{on} = 0.0001444 $nM^{-1}min^{-1}$. * negative value; [1] the antigen concentrations $[Ag]_0 = \{1.25; 2.5; 5; 10; 20; 40; 80\}$ nM are used; [2] the antigen concentrations $[Ag]_0 = \{2.5; 5; 10; 20; 40; 80\}$ nM are used; [3] the antigen concentrations $[Ag]_0 = \{5; 10; 20; 40; 80\}$ nM are used; [4] the antigen concentrations $[Ag]_0 = \{10; 20; 40; 80\}$ nM are used; [5] with hyperbola, failure to achieve equilibrium overestimates the K_D values. Even after exclusion of the bound fractions at $[Ag]_0 \leq 5$ nM, the K_D values are not exploitable; [6] Lindmo plot gives overestimated K_D values that become nonsensical when $K_D \geq 1$ nM. Even after excluding the bound fractions at $[Ag]_0 \leq 5$ nM, the K_D values are not exploitable.
